# Cuproptosis: Biomarkers, Mechanisms and Treatments in Diseases

**DOI:** 10.3390/molecules31030394

**Published:** 2026-01-23

**Authors:** Shuhui Wang, Jian Zhang, Yanyan Zhou

**Affiliations:** Department of Basic Medicine, Heilongjiang University of Chinese Medicine, Harbin 150040, China

**Keywords:** cuproptosis, oxidative stress, biomarker, signaling pathway, targeted therapy, natural product

## Abstract

The homeostasis balance of copper, as an essential trace element for life activities, is crucial for maintaining the normal function of cells. Cuproptosis, discovered in recent years, is a novel type of programmed cell death triggered by the accumulation of excessive copper ions in mitochondria. The core mechanism lies in that copper ions, after being reduced by ferridoxin (FDX1), directly target and induce the oligomerization of the acylated tricarboxylic acid (TCA) cycle enzyme, thereby triggering fatal protein toxic stress. This distinctive mechanism operates independently of other recognized pathways of cell death, offering a novel perspective for elucidating the pathological processes underlying various diseases. A review of pertinent research conducted over the past four years reveals that cuproptosis is not only significantly implicated in the onset, progression, and treatment resistance of tumors but is also intricately associated with diverse pathological processes, including neurodegenerative diseases, cardiovascular diseases, metabolic disorders, and immune abnormalities. This article conducts a multi-level summary from molecular mechanisms to physiological and pathological significance; deeply explores the interaction between cuproptosis and various subcellular structures, as well as their complex signal regulatory network; and systematically expounds the cutting-edge strategies for treating cuproptosis, including traditional copper chelating agents, ion carriers, and copper-based nanomedicines, with a particular focus on the latest progress in the field of natural product research. This review has systematically summarized the therapeutic potential demonstrated by numerous natural active ingredients when precisely regulating the cuproptosis pathway to provide a theoretical reference for future research in this field.

## 1. Introduction

Copper, as an indispensable trace metal element in the life system, plays a crucial physiological role in the human body [1]. It is widely distributed in key organs such as the liver, brain, and heart, with a total amount of approximately 110 milligrams. It is a core cofactor for multiple metalloenzymes, including cytochrome c oxidase, superoxide dismutase, and ceruloplasmin (CP), and is deeply involved in basic life processes such as cellular energy metabolism, antioxidant defense, and neurotransmitter synthesis [2,3,4,5]. The human body takes in copper through diet, mainly absorbed by the small intestine, and its excretion is precisely regulated by the liver and gallbladder system to maintain a dynamic balance [6]. However, once this balance is disrupted, whether due to genetic defects or environmental factors, leading to copper deficiency or excess, it will cause serious pathological consequences, including neurological degeneration, liver dysfunction, and developmental abnormalities [7,8,9].

At the cellular level, copper is one of several metal ions, including iron, zinc, and manganese, essential for sustaining life activities. The homeostasis of these metal ions contributes to the intricate intracellular microenvironment, which underpins normal physiological functions. The precise regulation of steady-state metals is crucial for their roles as catalytic centers, structural components, and signaling molecules [10]. The disruption of this homeostasis can result in cellular damage and may even lead to disease by inducing oxidative stress, disrupting metabolic pathways, and activating specific cell death programs. In recent years, the identification of a novel, copper-dependent programmed cell death mechanism—termed “cuproptosis”—illustrates the pathogenic processes associated with metal homeostasis imbalance and offers a new perspective for the treatment of related diseases [11]. Cuproptosis is characterized as a form of regulated cell death initiated by an excess of free copper ions within cells, particularly in mitochondria. Its occurrence is contingent upon active mitochondrial respiration. The fundamental mechanism involves the reduction of accumulated copper ions in the mitochondrial matrix by ferridoxin 1 (FDX1) to more reactive monovalent copper (Cu^+^), which directly interacts with metabolic enzymes modified by acylation in the tricarboxylic acid (TCA) cycle, such as dihydrolipoamide S-acetyltransferase (DLAT). This interaction induces abnormal oligomerization and the insoluble aggregation of these functional proteins while simultaneously disrupting the stability of iron–sulfur (Fe-S) cluster proteins [12,13,14,15]. This process ultimately induces severe protein-toxic stress, resulting in irreversible cell death. This pathway is resistant to inhibitors targeting other forms of cell death; however, it can be rescued by specific copper chelating agents, thereby establishing its distinct identity, separate from apoptosis, ferroptosis, and other mechanisms [16].

Although research on copper-induced cell death is extensive, most current reviews either concentrate on its molecular mechanisms or are confined to specific disease areas. Consequently, there is a lack of a systematic summary that integrates basic mechanisms, disease spectra, and treatment strategies, particularly in the emerging domains of natural products and nanomedicines. Based on this, we will start from the fundamental principles of copper homeostasis and regulation in the human body; we will systematically expound the occurrence mechanism of cuproptosis and its correlation with subcellular structures such as mitochondria and lysosomes. Then, we will elaborate its complex interaction network with multiple key signal pathways on a deep level. We subsequently summarize the biomarkers and pathogenic mechanisms associated with copper-induced cell death in various systemic diseases, including tumors, neurological disorders, cardiovascular conditions, digestive disorders, and immune system dysfunctions. Finally, we conduct a comprehensive review of drug treatment strategies aimed at targeting copper-induced mortality, encompassing both traditional chemical agents and innovative nanomedicines. Notably, we systematically organize the recent research advancements and mechanistic analyses regarding the regulation of copper-induced mortality by natural products. This retrospective study aims to furnish researchers in the field with a clear and comprehensive knowledge framework. It seeks to deepen the understanding of the biological significance of cuproptosis while also providing valuable insights and references for future investigations into cuproptosis pathways, biomarker discovery, and innovative drug design.

## 2. Copper in the Human Body

Copper, an essential trace metal in biological systems, is widely distributed across various tissues and organs in the human body, totaling approximately 110 milligrams, and serves an irreplaceable physiological function. Copper predominantly exists in two oxidation states: monovalent copper (Cu^+^) in the reduced state and divalent copper (Cu^2+^) in the oxidized state. Under physiological conditions, it can engage in REDOX reactions by either accepting or donating electrons. This property renders it an essential cofactor for numerous key enzymes [17,18]. The distribution of copper in the human body shows obvious tissue heterogeneity. The liver, as the central organ for copper homeostasis, has a reserve of approximately 10 mg. It is not only the main storage site for copper but also undertakes the regulatory functions of copper redistribution and excretion [19]. The brain is the organ with the second-highest copper content, at approximately 8.8 milligrams. The concentration is particularly significant in regions related to cognition, movement, and emotion regulation, such as the substantia nigra, hippocampus, and cortex, suggesting that copper plays a special role in the nervous system [20]. The heart possesses a relatively high concentration of copper, which is closely associated with the elevated energy demands and oxidative metabolism of myocardial cells. While the total copper content in muscles and bones is substantial, the concentration per unit is comparatively low due to the significant tissue mass. In the circulatory system, copper is primarily transported in conjunction with proteins. The copper content in whole blood is approximately 6 mg, with about 1 mg present in plasma. In this section, approximately 65% of the copper is firmly bound to ceruloplasmin in a covalent manner, 18% is bound to albumin, and 12% is bound to transferrin, while the remaining 5% forms exchangeable pools with small molecule ligands such as glutathione. The copper in this pool has high bioavailability and can quickly respond to the metabolic needs of tissues [21].

**Figure 1:**
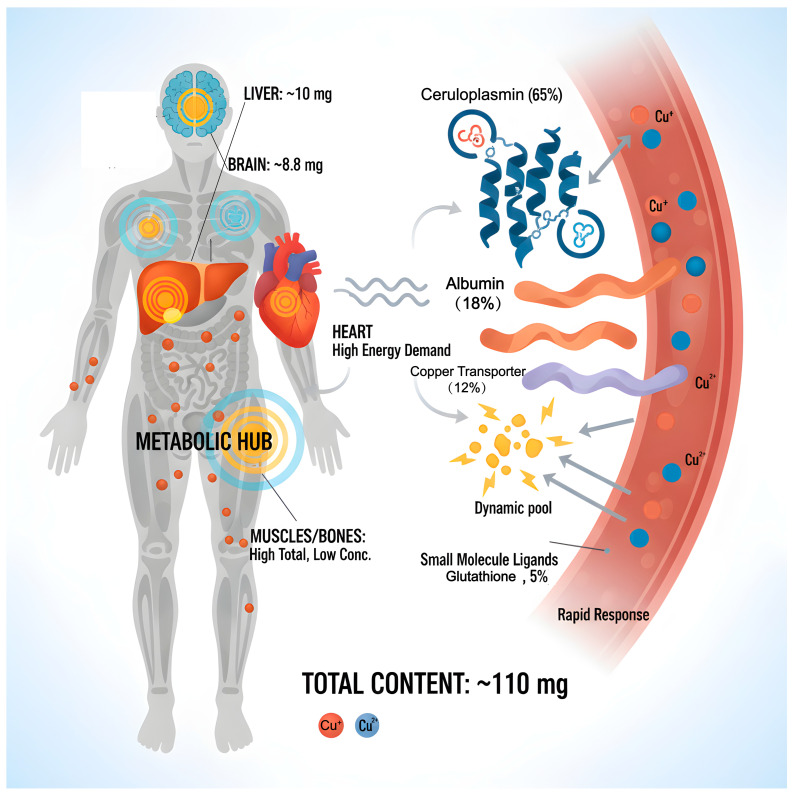
The distribution of copper in the human body. The total copper content in the human body is approximately 110 mg, distributed across various tissues with significant heterogeneity. The liver (about 10 mg) serves as the central organ for copper homeostasis, responsible for storage and distribution; the brain (about 8.8 mg) is enriched in regions associated with cognition and motor function, highlighting its role in neural regulation; the heart also contains high copper levels due to its high energy demands. In the circulatory system, the total blood copper content is about 6 mg, with plasma copper primarily bound to ceruloplasmin (65%), albumin (18%), and transcuprein (12%), while the remaining 5% forms a bioavailable exchangeable pool with small ligands such as glutathione. As an essential cofactor, copper participates in core physiological processes such as cellular energy metabolism, antioxidant defense, and neurotransmitter synthesis through its redox properties. The dysregulation of copper homeostasis is closely associated with neurodegenerative diseases.

Copper’s biological functions primarily manifest through its role as a metal cofactor in catalyzing diverse biochemical reactions. As an essential component of cytochrome c oxidase (CCO), which resides in the inner mitochondrial membrane, it acts as the terminal enzyme in the oxidative phosphorylation pathway. CCO facilitates the transfer of electrons to oxygen and promotes ATP synthesis, thereby playing a central role in cellular energy metabolism [22]. Copper–zinc superoxide dismutase (SOD1) is activated by copper and can catalyze the conversion of superoxide anions to hydrogen peroxide. It is an important antioxidant defense enzyme in cells, protecting cells from oxidative damage [23,24,25]. Ceruloplasmin functions as the principal copper transport protein in plasma and exhibits iron oxidase activity, which enables the oxidation of divalent iron to trivalent iron, thereby facilitating the binding and utilization of iron by transferrin. Consequently, it plays a crucial role in maintaining iron metabolic homeostasis [26,27,28]. Furthermore, copper serves as an essential component of numerous functional enzymes. Tyrosinase plays a critical role in melanin biosynthesis, thereby influencing skin and hair pigmentation [29]. Lysyl oxidase facilitates the cross-linking of collagen and elastin, which is vital for preserving the mechanical integrity of connective tissue [30]. Additionally, dopamine β-hydroxylase catalyzes the conversion of dopamine to norepinephrine, impacting neural transmission and stress responses [31]. Beyond its enzymatic roles, copper plays a significant part in various physiological processes, including angiogenesis, immune cell activation, myelin formation, and neurotransmitter synthesis. Recent studies have demonstrated that copper can affect cell proliferation, differentiation, and apoptosis by modulating the MEK/ERK and Notch signaling pathways, thereby assuming a dual role in tumorigenesis and development [32,33]. As a copper-rich organ, the brain’s dysregulation of copper homeostasis is closely associated with several neurodegenerative diseases, including Alzheimer’s disease, Parkinson’s disease, and Huntington’s disease. This relationship underscores the profound physiological and pathological significance of copper in preserving the function and integrity of the nervous system (Figure 1).

## 3. Copper Homeostasis

**Figure 2:**
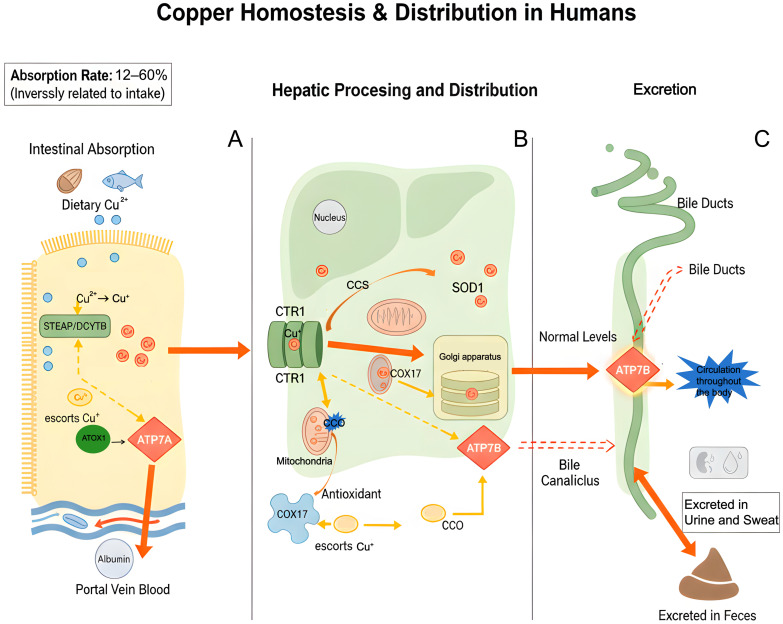
Copper homeostasis within the human body. (**A**) The Cu^2+^ in the diet is reduced to Cu^+^ within the intestinal lumen through the STEAP/DCYTB enzyme on the brush border membrane. The partner protein ATOX1 transports Cu^+^ to the trans-Golgi network and is then pumped into the portal vein circulation by ATP7A. (**B**) The copper in the portal vein blood (bound to albumin/transcuprin) enters the liver cells through the CTR1 channel. Copper is delivered to SOD1 by CCS and exerts antioxidant functions. Copper is transported by COX17 to the mitochondria and enters cytochrome c oxidase (CCO) to ensure the function of the respiratory chain. When copper is excessive, ATP7B translocates to the bile duct membrane and pumps copper into the bile for excretion. (**C**) Copper is mainly excreted through the bile pathway of the liver. A very small amount of copper can be excreted through urine, sweat, feces and other means.

Copper homeostasis in the human body represents a complex system that coordinates multiple organs and proteins. This process includes the absorption of dietary copper, its transport in the bloodstream, intracellular distribution and utilization, and eventual excretion. Each step is meticulously regulated to ensure overall homeostasis. Dietary copper primarily originates from sources such as animal offal, seafood, nuts, legumes, and whole grains, typically existing in the form of Cu^2+^ [34]. Copper absorption primarily takes place in the proximal segment of the small intestine, particularly within the duodenum and jejunum, through passive transport mechanisms, including transmembrane movement mediated by specific transport proteins and binding to carrier proteins [35]. Initially, metal-reductases situated on the brush border membrane of intestinal epithelial cells, such as STEAP family proteins and duodenal cytochrome b (DCYTB), facilitate the reduction of Cu^2+^ to Cu^+^. The resulting Cu^+^ serves as the appropriate substrate for high-affinity copper transporter 1 (CTR1/SLC31A1) [36,37]. CTR1 functions as a trimer and facilitates the transport of Cu^+^ into cells via conformational changes. When CTR1 is absent or its function is compromised, divalent metal transporter 1 (DMT1) can serve as a compensatory mechanism to mediate the influx of Cu^2+^. The efficiency of copper absorption is inversely related to the amount ingested [38,39]. Under a low-copper diet, the absorption rate can exceed 60%; however, when dietary copper is excessive, the absorption rate may decline to approximately 12%. This adaptive regulation effectively prevents the accumulation of excess copper [40]. Upon entering intestinal cells, copper undergoes further processing via multiple pathways. A fraction of copper is temporarily sequestered in the cytoplasm through binding with metallothionein (MT) or glutathione (GSH). Another portion is transported to the trans-Golgi network (TGN) through its association with the copper transporter antioxidant 1 (ATOX1), subsequently being released into the portal venous circulation from the basolateral membrane, a process mediated by ATP7A [41]. Copper in portal vein blood primarily binds to albumin and transferrin before being transported to the liver. Upon uptake by liver cells via CTR1, copper can be integrated into newly synthesized ceruloplasmin or transferred to intracellular chaperone proteins for further distribution. The copper chaperone for superoxide dismutase 1 (CCS) facilitates the delivery of copper to SOD1, thereby enhancing its antioxidant activity. Cytochrome c oxidase copper chaperone 17 (COX17) transports copper into the mitochondrial membrane space and aids in its incorporation into CCO through the action of cytochrome c oxidase 1 (SCO1), SCO2, or COX11, which is essential for the proper functioning of the mitochondrial respiratory chain. Conversely, ATOX1 is tasked with delivering copper to ATP7A and ATP7B, which are critical for the maturation of copper-dependent enzymes such as tyrosinase and lysyl oxidase, as well as for the transmembrane transport of copper [42,43,44,45]. The liver serves as the primary organ responsible for regulating copper homeostasis. At normal copper concentrations, ATP7B facilitates the incorporation of copper into ceruloplasmin, subsequently releasing it into the bloodstream for distribution to various body tissues. In conditions of copper excess, ATP7B relocates to the bile duct membrane, where it secretes copper into bile, leading to its excretion from the body via feces [46]. Moreover, a minimal quantity of copper can be excreted via urine, sweat, and other pathways. This intricate metabolic network not only facilitates the efficient utilization of copper but also sustains the dynamic balance of copper concentration in the body through a dual mechanism involving liver and gallbladder excretion and absorption regulation (Figure 2).

## 4. Copper Regulation

The maintenance of copper homeostasis in the human body relies on a complex and dynamic regulatory network that encompasses transcriptional regulation, post-translational modifications of proteins, alterations in subcellular localization, and system-level regulation. Disruptions in any of these components can result in physiological dysfunction and may contribute to the development of diseases. At the transcriptional level, the expression of multiple copper-related genes is precisely regulated by intracellular copper concentration, including CTR1, Metallothionein (MT), and nuclear factor erythroid 2-related factor 2 (Nrf2). The promoter region of the copper transporter CTR1 features a GC cassette that binds the transcription factor Sp1 [47]. In low-copper conditions, Sp1 is activated, which promotes CTR1 transcription and enhances copper uptake. Conversely, when copper levels are high, Cu^2+^ binds directly to Sp1, inhibiting its DNA binding and downregulating CTR1 expression, thus establishing a negative feedback loop [48]. The expression of metallothionein (MT) is regulated by the metal response element (MRE) and its binding factor, metal transcription factor 1 (MTF-1). In conditions of copper excess, MTF-1 translocates to the nucleus, where it initiates MT transcription and chelates free copper through its cysteine-rich domain, thereby mitigating cytotoxicity [49]. Additionally, the antioxidant transcription factor Nrf2 is activated in response to copper stress, leading to the upregulation of a series of antioxidant genes, including NAD(P)H dehydrogenase [quinone] 1 (NQO1), superoxide dismutase (SOD), glutathione S-transferase (GST), and heme oxygenase 1 (HO-1), which collectively assist in maintaining REDOX homeostasis [50].

At the level of protein function and subcellular localization, copper chaperones and transporters facilitate the directional delivery and distribution of copper via specific protein–protein interactions. The cytoplasmic chaperone ATOX1 binds Cu^+^ through its CXXC motif and transports it to ATP7A and ATP7B within the TGN [51]; These P-type ATPase enzymes are situated at the TGN under basal copper conditions and facilitate the loading of copper onto ceruloplasmin. When the intracellular copper concentration rises, ATP7A/B experiences phosphorylation and subsequently translocates to the cell membrane, with ATP7A relocating to the basolateral side and ATP7B moving to the apical membrane, thereby directly mediating copper efflux [52]. Copper transport within mitochondria is mediated by the COX17-SCO1/2-COX11 pathway, which facilitates accurate copper insertion into the CuA and CuB sites of CCO. Disruptions in this pathway may result in mitochondrial dysfunction and the accumulation of reactive oxygen species (ROS) [53]. Furthermore, copper can influence signaling pathways by modulating protein–protein interactions. It can directly bind to MEK1/2, thereby enhancing its kinase activity and promoting the activation of the ERK signaling pathway, which subsequently affects cell proliferation and survival [54]. In tumor cells, the overexpression of copper chaperones, such as CCS and ATOX1, can facilitate tumor growth and invasion through these mechanisms.

At the system level, the maintenance of copper homeostasis requires the coordinated efforts of multiple organs. The liver serves as a primary regulatory organ, facilitating the bidirectional regulation of copper balance throughout the body via the synthesis of ceruloplasmin and the excretion of bile. The intestine modulates the efficiency of copper absorption by regulating the expression of CTR1 and ATP7A. Furthermore, the blood–brain barrier (BBB) and the blood–cerebrospinal fluid barrier (BCB) meticulously control copper entry and exit from the brain through the expression of transporters such as CTR1, ATP7A, and DMT1, thereby sustaining copper homeostasis in the central nervous system [55]. During inflammatory or infectious conditions, the liver’s capacity to synthesize ceruloplasmin is upregulated, resulting in increased serum copper levels. This response may serve as a host defense mechanism that either limits the availability of copper to microorganisms or enhances the functionality of immune cells [56]. Under pathological conditions, abnormalities in the copper regulatory network are evident through the dysregulation and mutation of copper transporters. For example, mutations in ATP7B result in Wilson’s disease, characterized by abnormal copper accumulation in tissues such as the liver, brain, and cornea, leading to liver cirrhosis and neuropsychiatric manifestations. Conversely, mutations in ATP7A give rise to Menkes disease, which is marked by impaired copper absorption and systemic copper deficiency. This deficiency disrupts the function of various copper-dependent enzymes, ultimately resulting in developmental delays and neurodegeneration [57,58].

## 5. Cuproptosis

### 5.1. The Basic Mechanism of Cuproptosis Occurrence

**Figure 3:**
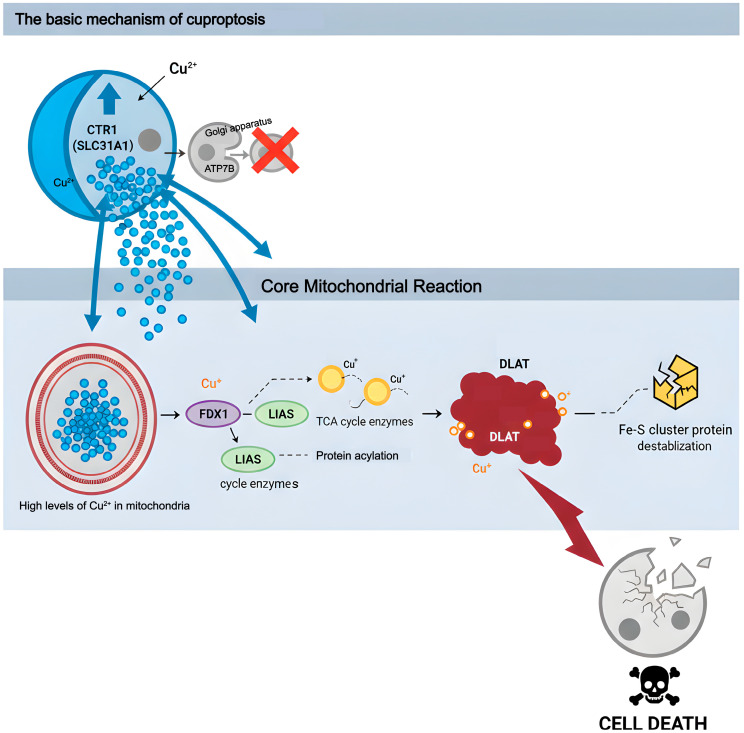
The basic mechanism of cuproptosis. When the main copper uptake protein CTR1 (SLC31A1) on the cell membrane is overexpressed, while the key protein ATP7A/B responsible for copper excretion is functionally impaired, a large amount of Cu^2+^ accumulates abnormally within the cells and forms high-concentration aggregations in the mitochondria. In the mitochondrial matrix, the FDX1 protein reduces Cu^2+^ to a more reactive form of Cu^+^. Simultaneously, the LIAS protein catalyzes the lipid acylation modification of DLAT in the TCA cycle. Subsequently, Cu^+^ specifically targets and binds to these lipid-acylated DLAT proteins, inducing abnormal intermolecular cross-linking and oligomerization, and ultimately forming insoluble protein aggregates. This abnormal protein aggregation not only directly damages the ultrastructure of mitochondrial cristae, but also leads to the destabilization and loss of function of key Fe-S cluster proteins in the mitochondria, thereby severely impairing the function of the electron transfer chain and energy metabolism.

Cuproptosis is a recently identified and highly regulated form of cell death that represents a type of programmed cell death induced by the excessive accumulation of copper ions within cells. The primary molecular mechanism underlying this process takes place within the mitochondrial matrix. FDX1, located in the mitochondrial matrix, not only reduces Cu^+^ to a more reactive form but also acts as a crucial upstream promoter of protein acylation [59,60,61]. Lipoate synthase (LIAS) serves as an essential cofactor for the catalysis of protein lipoylation. Consequently, FDX1 and LIAS together establish the molecular foundation for the phenomenon known as cuproptosis. The reduced Cu^+^ directly targets and binds to the acylating metabolic enzymes modified by LIAS in the tricarboxylic acid (TCA) cycle, with DLAT being the most prominent marker. The interaction between copper-acylated proteins promotes abnormal oligomerization and the insoluble aggregation of proteins, including DLAT, resulting in functional loss. Concurrently, this interaction severely disrupts the stability and biosynthesis of iron–sulfur (Fe-S) cluster proteins [62]. Ultimately, these events collectively induce irreversible protein-toxic stress, culminating in cell death. This death pathway is highly dependent on active mitochondrial respiration and cannot be reversed by inhibitors of other cell death types. However, it can be salvaged by specific copper chelators, highlighting the unique molecular characteristics of cuproptosis as an independent cell death modality (Figure 3).

### 5.2. Subcellular Structures Related to Cuproptosis

**Figure 4:**
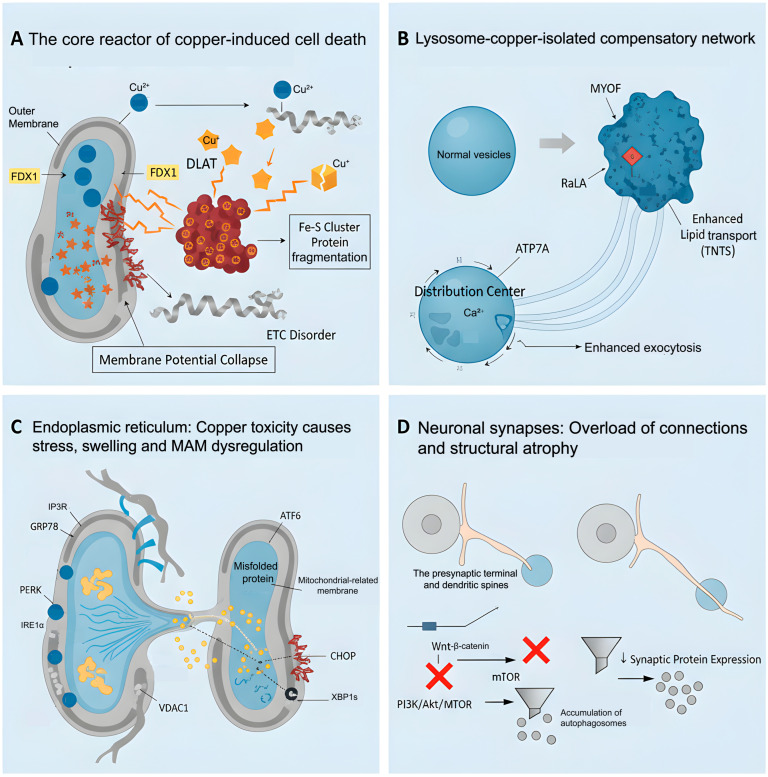
The influence of cuproptosis on subcellular structures. (**A**) Mitochondria are the core reactors of copper-induced death. Excessive Cu^2+^ entering the matrix is reduced to Cu^+^ by FDX1, directly damaging the mitochondrial cristae structure and causing fragmentation of Fe-S cluster proteins and disruption of the electron transport chain, ultimately leading to membrane potential collapse and energy metabolism failure. (**B**) The lysosome performs compensatory isolation function. ATP7A pumps excessive Cu^2+^ into the lumen, while membrane proteins MYOF/RalA are upregulated to enhance lipid transport, promoting the formation of tunnel nanotubes (TNTs), transforming the lysosome from an isolated vesicle to a networked copper distribution center, with its morphology becoming irregular and the exocytosis activity enhanced. (**C**) The endoplasmic reticulum experiences severe stress and structural disorder. Copper toxicity leads to the accumulation of misfolded proteins in the lumen, triggering stress markers GRP78, PERK, IRE1α and ATF6, and causing the endoplasmic reticulum cavity to swell and tend to fragment; simultaneously, the IP3R-VDAC1 connection on the mitochondrial-associated membrane (MAM) is disrupted and calcium homeostasis is disrupted, further activating downstream stress pathways such as CHOP/XBP1s. (**D**) The neuronal synapse undergoes structural atrophy due to the inhibition of key pathways. Copper toxicity simultaneously inhibits the Wnt/β-catenin pathway and the PI3K/Akt/mTOR pathway, both of which synergistically result in a reduction in the number of dendritic spines, the atrophy of synaptic spines, and the deformation of the neurite.

Cuproptosis, a newly identified copper-dependent mode of cell death, is intricately linked to the morphological and functional alterations of essential subcellular structures, including mitochondria, lysosomes, the endoplasmic reticulum, and synapses. Within mitochondria, excess copper ions are transported to the matrix through ion carriers and are reduced to highly reactive Cu^+^ under the catalytic influence of FDX1. Cu^+^ ions specifically bind to the lipoacyl groups in acylated TCA cycle enzymes, initiating the formation of intermolecular disulfide bonds. This process results in the abnormal oligomerization of these metabolic enzymes and the subsequent formation of insoluble aggregates. Such protein aggregation not only directly disrupts the normal ultrastructure of mitochondrial cristae, leading to the swelling and disintegration of the cristae structure, but also impairs the function of the electron transport chain by depleting key metabolic enzymes and destabilizing F-S cluster proteins. Ultimately, this cascade of events results in the collapse of membrane potential and failure of energy metabolism [63,64]. Lysosomes serve as the primary center for degradation and recycling within cells, participating in the decomposition of biological macromolecules, including proteins and lipids. Additionally, they play a crucial role in the isolation and detoxification of metal ions. By chelating and sequestering copper, lysosomes can directly influence copper homeostasis, thereby reducing cytotoxicity [65]. The ATP7A transporters actively transport excess copper ions into the lysosomal chamber for sequestration, while simultaneously upregulating the expression of membrane proteins such as MYOF and RalA. This process enhances lipid transport capacity and promotes the formation of tunnel nanotubes [66]. Consequently, these modifications have redefined lysosomes from isolated degradation organelles into interconnected centers for copper ion distribution. Additionally, their morphology has adapted, exhibiting changes in membrane surface properties and increased exocytotic activity. The endoplasmic reticulum (ER) is a crucial organelle involved in protein synthesis, folding, modification, and calcium ion storage. The stability of its internal environment underpins cellular function, while alterations in its morphology and function resulting from copper-induced cell death are particularly intricate. Copper exposure markedly upregulates key indicators of endoplasmic reticulum (ER) stress, including GRP78/BiP, PERK, ATF6, IRE1α, and its downstream products CHOP and XBP1s. The intense oxidative stress induced by excessive copper directly disrupts the oxidative environment within the ER, inhibits the proper formation of disulfide bonds, and results in protein misfolding. Consequently, this process leads to the significant dilation of the endoplasmic reticulum and swelling of the cystic cavities [67,68,69]. Copper poisoning frequently results in mitochondrial dysfunction and reduced ATP synthesis. The process of protein folding is highly energy-intensive, and an energy deficit will inevitably exacerbate the burden of protein folding within the endoplasmic reticulum (ER). The ER is intricately linked to mitochondria via the mitochondrial-associated endoplasmic reticulum membrane (MAM) structure, which facilitates the exchange of lipids, calcium ions, and signaling molecules. A weakened interaction between IP3R and VDAC1 disrupts calcium homeostasis, further intensifying ER stress and promoting the sustained activation of the PERK and IRE1α signaling pathways. This cascade ultimately leads to ER fragmentation and functional disintegration [70]. The synapse is a critical structure for information transmission between neurons, facilitating the conversion and modulation of electrical and chemical signals. It also serves as the microscopic foundation for brain function and cognitive activities. In neuronal synapses, copper toxicity impacts their structure through a dual mechanism. First, the excessive accumulation of Cu directly inhibits the Wnt/β-catenin pathway, leading to a decrease in the transcriptional expression of synaptic-associated proteins. Second, it disrupts the PI3K/Akt/mTOR pathway, resulting in autophagic flow blockade and the accumulation of damaged organelles. The synergistic effects of these molecular events ultimately lead to a reduction in the number of synapses, the atrophy of synaptic spines, and neural mutagenesis (Figure 4).

### 5.3. Pathways Related to Cuproptosis

#### 5.3.1. TCA Cycle

The TCA cycle, central to cellular energy metabolism, features key enzymes, including DLAT and dihydrolipoamide succinyltransferase (DLST), which serve as essential executors in the process of copper-induced cell death. Copper ions can directly interact with the thioacyl groups in acylating enzymes, resulting in irreversible oligomerization reactions that induce protein toxic stress within mitochondria and ultimately lead to cell death. The Cu(L1)_2_Cl complex effectively depletes intracellular glutathione (GSH), releases copper ions, and increases reactive oxygen species (ROS) generation, thereby facilitating the aggregation of DLAT. This finding confirms the direct involvement of TCA cycle enzymes in copper-induced cell death [71]. It is particularly worth noting that in colon cancer studies, cuproptosis was found to be accompanied by changes in the lactate/pyruvate ratio, suggesting a bidirectional regulatory relationship between metabolic reprogramming and cuproptosis. Therefore, the TCA cycle is not only the center of energy metabolism but also transforms into a “death signal amplifier” under copper overload conditions.

#### 5.3.2. Nrf2 Pathway

The Nrf2-Keap1-ARE pathway constitutes the core cellular antioxidant defense system. Copper ion overload disrupts the canonical Keap1-Cul3-E3 ubiquitin ligase-mediated degradation of Nrf2, likely by affecting its N-terminal Neh2 domain, thereby stabilizing Nrf2 and inducing the expression of cellular antioxidant enzymes. The activation of Nrf2 exhibits a dual role in copper-induced cell death. Specifically, it can mitigate copper-induced oxidative damage by upregulating antioxidant genes. Conversely, it may also facilitate copper excretion by modulating the copper transporters ATP7A and ATP7B, thereby increasing cellular resistance to copper-induced death. Circular RNA circSpna2 binds to the DGR domain of Keap1, inhibiting the ubiquitination and degradation of Nrf2. This interaction enhances the transcription of the *ATP7B* gene and significantly mitigates copper-induced cell death in nerve cells. The identification of this non-coding RNA-Nrf2-Cu axis underscores the critical role of post-transcriptional regulation in the context of copper-induced cell death. The discovery of meritinib (MTB) further underscores the central role of the Nrf2 pathway. MTB diminishes reactive oxygen species (ROS) activity by directly binding to the Nrf2 protein. Additionally, it inhibits elesclomol-CuCl2-mediated copper influx and the aggregation of acylated proteins and iron–sulfur (Fe-S) cluster proteins. This mechanism promotes Cu^2+^ efflux, thereby achieving a dual inhibitory effect on copper-induced cell death [72].

#### 5.3.3. HIF-1α Pathway

Hypoxia-inducible factor 1α (HIF-1α) serves as a central regulatory element in cellular adaptation to hypoxic conditions and demonstrates a surprisingly diverse array of functions in the regulation of copper-induced cell death. HIF-1α influences sensitivity to cuproptosis through various mechanisms. In models of myocardial infarction, dapagliflozin significantly decreases Cu^2+^ accumulation and reactive oxygen species (ROS) generation by inhibiting the HIF-1α/TGF-β signaling pathway and downregulating the copper transporter CTR1, which enhances copper uptake and mitigates copper-induced myocardial fibrosis [73]. In tumors, the role of HIF-1α exhibits tissue specificity. The overexpression of VIPR1 increases the sensitivity of colon cancer cells to copper-induced cell death by inhibiting HIF-1α signaling [74]. Conversely, in triple-negative breast cancer, HIF-1α may indirectly inhibit FDX1 through the activation of AKT signaling, leading to resistance to copper-induced cell death [75]. This discrepancy indicates that the regulation of copper-induced cell death by HIF-1α is context-dependent and may be influenced by cell type, metabolic status, and microenvironmental factors.

#### 5.3.4. PI3K Pathway

The phosphatidylinositol-3-kinase (PI3K) pathway, recognized as a classic cell survival signaling network, primarily exerts an inhibitory effect on the regulation of copper-induced cell death. Numerous studies have collectively shown that the activation of this pathway enhances cellular resistance to cuproptosis through various mechanisms. Protein kinase B (AKT/PKB), a key downstream target of PI3K, directly phosphorylates FDX1, thereby inhibiting its function and promoting glycolytic metabolic reprogramming, which ultimately reduces sensitivity to copper-induced cell death. In the osteonecrosis model, it was observed that the PI3K/AKT pathway influences the process of cuproptosis through the regulation of FDX1 expression. The PI3K agonist 740Y-P is capable of restoring FDX1 levels and mitigating Cu^2+^ aggregation in osteoblasts [76]. A separate study demonstrated that the downregulation of FDX1 expression in hepatocellular carcinoma facilitates tumor tissue infiltration and growth. Enhancing FDX1 expression significantly inhibits the progression of HCC and oral squamous cell carcinoma (OSCC) by influencing metabolic reprogramming, elevating ROS levels, and activating mitochondrial autophagy and the PI3K/AKT pathway. Consequently, FDX1 may serve as a potential prognostic biomarker and therapeutic target [77]. mTOR serves as a critical activation target downstream of AKT. CCAAT/enhancer-binding protein beta (CEBPB) enhances cell migration and proliferation in colorectal cancer tissues by activating the PI3K/AKT/mTOR pathway, while simultaneously reducing levels of copper-induced cell death [78]. Furthermore, glycogen synthase kinase-3 β (GSK-3β), when activated, can enhance the stable expression of activating transcription factor 3 (ATF3). This transcription factor directly upregulates the expression of the copper transporter CTR1, resulting in intracellular copper ion overload and subsequent copper-induced cell death [79].

#### 5.3.5. ATOX1 Pathway

ATOX1, a crucial protein involved in intracellular copper transport, serves functions that extend beyond its role as a metal chaperone. It binds copper ions via its MXCXXC motif, facilitating the formation of a homodimer or interacting with the N-terminal domain of ATP7A/ATP7B, which is responsible for transporting copper ions from the cell membrane to the Golgi network [80]. In diffuse large B-cell lymphoma (DLBCL), ATOX1 expression is significantly elevated. The persistent activity of the mitogen-activated protein kinase (MAPK) pathway promotes cell proliferation, whereas the knockdown of ATOX1 induces G2/M phase arrest and increases sensitivity to copper-induced cell death. This suggests that ATOX1 may indirectly influence copper-induced cell death by modulating the cell cycle process [81]. ATOX1 possesses a nuclear localization function and can directly regulate the expression of genes such as CCND1, SOD3, and NCF1 as a transcription factor [82]. Notably, the nuclear-cytoplasmic shuttling ability of ATOX1 may serve as a critical switch in the regulation of copper-induced cell death. Within the nucleus, ATOX1 may enhance cellular resistance to copper toxicity by modulating the expression of antioxidant genes. Conversely, in the cytoplasm, it plays a direct role in the distribution and transport of copper ions.

#### 5.3.6. cGAS Pathway

The circular GMP-AMP synthase (cGAS) pathway serves as a fundamental component of innate immunity, with its activation initiated by the presence of abnormal viral, bacterial, or self-derived double-stranded DNA in the cytoplasm. cGAS identifies and binds to these DNA molecules, leading to its activation and the synthesis of the second messenger molecule 2′3′-cGAMP. Following this, cGAMP interacts with the stimulator of interferon genes (STING) located on the endoplasmic reticulum, resulting in conformational changes in STING and its subsequent transport from the endoplasmic reticulum to the Golgi apparatus [83]. During this process, STING undergoes polymerization and recruits the kinase TBK1, which subsequently phosphorylates the transcription factor IRF3. This phosphorylation results in the formation of a dimer that translocates to the nucleus, where it initiates the expression of type I interferons and various pro-inflammatory cytokine genes. Cuproptosis induces mitochondrial dysfunction and the release of mtDNA, thereby activating the cGAS-STING signaling cascade, which promotes intense immunogenic cell death (ICD) and robust anti-tumor immune responses [84]. The pLCGM-OVA nano-immune agonist exemplifies the therapeutic potential of this mechanism. This nanoparticle releases Cu^2+^ within the tumor microenvironment to induce cuproptosis, while the released Mn^2+^ enhances the activation of cGAS-STING, resulting in a synergistic anti-tumor effect. Zinc-doped copper sulfide nanoflowers (ZCS NFs) not only induce cuproptosis but also release Zn^2+^, which synergizes with mtDNA to activate the STING pathway, thereby achieving a “dual-signal” activation mode. Additionally, both Cu-DPPZ-Py and Elesclomol-induced cuproptosis significantly enhanced mtDNA release and cGAS-STING activation, whereas the apoptosis inducer Cu-DPPZ-Ph did not exhibit this effect.

#### 5.3.7. COMMD1 Pathway

COMMD1 is a multifunctional scaffold protein characterized by the presence of a COMM domain at its C-terminus. This domain interacts with ATP7B to regulate copper homeostasis and facilitate copper excretion. Additionally, COMMD1 negatively modulates inflammatory responses by binding to the NF-κB subunit p65, thereby promoting its ubiquitination and subsequent degradation [85]. Additionally, it plays a role in inhibiting the activity of hypoxia-inducible factor HIF-1α and regulating the intracellular transport of various membrane proteins. In the neonatal hypoxic–ischemic encephalopathy (HIE) model, hypoxic stress disrupts the COMMD1/ATP7A signaling axis, leading to the abnormal accumulation of Cu2+ and copper-induced cell death. This process results in increased reactive oxygen species (ROS) and decreased superoxide dismutase (SOD), which are directly associated with neuronal damage and cognitive dysfunction [86]. This finding is significant as it establishes a direct link between copper homeostasis disorders and the pathological mechanisms underlying perinatal brain injury, thereby offering a novel target for HIE treatment.

#### 5.3.8. C5a Pathway

The C5a/C5aR pathway constitutes a fundamental inflammatory signaling mechanism activated by the complement system. Upon activation by pathogens or tissue injury, C5, a crucial component of the complement cascade, is cleaved to produce the potent inflammatory mediator C5a. C5a functions as a chemokine, exhibiting high-affinity binding to the G protein-coupled receptor C5aR1, which is extensively expressed on the surface of myeloid immune cells [87]. This interaction triggers intracellular calcium ion mobilization, actin reorganization, and the activation of MAPK pathways, including PI3K. Studies have demonstrated that the activation of the C5a/C5aR pathway in breast cancer cells upregulates ATP7B expression via the Wnt/β-catenin signaling axis, thereby enhancing copper excretion capacity and conferring resistance to copper-induced cell death [88]. The combination of CuS nanoparticles, which induce cuproptosis, with C5a receptor antagonists (C5aRA) can restore tumor cell sensitivity to cuproptosis by inhibiting the C5a/C5aR signaling pathway, significantly augmenting the anti-tumor effect.

#### 5.3.9. SPI1 Pathway

SPI1, or SPI1, is a transcription factor that is essential for the development of the hematopoietic system. Its protein structure features a C-terminal ETS domain, which facilitates specific recognition and binding to DNA. Additionally, an N-terminal transcriptional activation domain is responsible for recruiting co-activators that initiate gene transcription [89,90]. SPI1 serves as a “master regulator” critical for the development of myeloid cells and B lymphocytes, with its expression level influencing cell differentiation in a dose-dependent manner. The SPI1/CDKN2A/p53 signaling axis is significant in regulating copper-induced mortality in lung adenocarcinoma. Specifically, the downregulation of the transcription factor SPI1 in LUAD results in diminished transcriptional inhibition of CDKN2A, leading to increased CDKN2A expression and the subsequent activation of the p53 signaling pathway. This cascade ultimately heightens the sensitivity of cells to copper-induced death [91]. The identification of this linear regulatory relationship is highly significant, as it links the tumor suppressor gene network to the cuproptosis process. Both the overexpression of SPI1 and the knockdown of CDKN2A can enhance cuproptosis induced by elesclomol-CuCl_2_, while the copper chelator TTM can reverse these effects, thereby demonstrating the specificity of cuproptosis within this pathway. Notably, copper treatment itself can feedback regulate this pathway. Copper-induced cuproptosis is associated with an upregulation of SPI1 and a downregulation of CDKN2A, creating a positive feedback loop that may represent an adaptive mechanism by which cells respond to copper stress.

#### 5.3.10. ERK Pathway

The ERK/MAPK signaling pathway serves as a fundamental regulatory network in the cellular stress response and plays a multifaceted role in copper-induced cell death. Research has demonstrated that nanoplastics (PS-NPs) induce oxidative stress and activate the ERK-MAPK pathway, which results in copper accumulation and subsequent neuronal death; notably, the MAPK inhibitor PD98059 can effectively reverse this process [92]. This finding suggests that, in certain contexts, the activation of the ERK/MAPK pathway may act as an upstream mediator of copper-induced cell death. Investigations in hepatocellular carcinoma have revealed that SEC14L3 regulates copper-induced cell death through the ERK/YY1/FDX1 axis, further underscoring the critical role of ERK signaling in this process [93]. Particularly noteworthy is the observation in diffuse large B-cell lymphoma (DLBCL) that ATOX1 knockdown results in reduced phosphorylation levels of ERK1/2, while treatment with CuCl_2_ can restore ERK activity, indicating that copper ions may influence cell fate by modulating the MAPK pathway [94].

#### 5.3.11. Wnt/β-Catenin Pathway

The Wnt/β-catenin signaling pathway is well recognized for its crucial role in embryonic development and tissue homeostasis; however, recent studies have innovatively elucidated its functional connection to cuproptosis. Cuproptosis activates the Wnt/β-catenin pathway, and the subsequent abnormal activation of this pathway enhances copper excretion by inducing ATP7B expression, thereby establishing a negative feedback regulatory loop that bolsters the resistance of cancer stem cells (CSC) to cuproptosis [95]. This finding highlights a sophisticated strategy employed by tumor cells to leverage developmental signaling pathways for evading metal stress. Cu^2+^ directly binds to PDK1, promoting its interaction with AKT and consequently activating the β-catenin signal. The β-catenin/TCF4 complex binds to the ATP7B promoter to induce its expression, thereby completing the adaptive response against cuproptosis. Research in breast cancer further supports this mechanism, as the C5a/C5aR pathway upregulates ATP7B expression via Wnt/β-catenin signaling, resulting in increased resistance to cuproptosis [96]. Thus, the Wnt/β-catenin pathway functions as an “intelligent regulator” in the context of cuproptosis regulation. It can be activated by cuproptosis while simultaneously inhibiting it through the enhancement of copper detoxification mechanisms. This self-limiting regulatory mode may represent a critical mechanism by which cells maintain metal homeostasis (Table 1).

## 6. Biomarkers and Mechanisms of Copper Mortality in Diseases

### 6.1. Tumor

The occurrence and development of tumors is a complex process driven by multiple genetic and epigenetic alterations, with core features including continuous proliferation, resistance to cell death, metabolic reprogramming, and immune escape [97]. During tumorigenesis and progression, the dysregulation of copper homeostasis forms a co-dynamic pathological cycle with the malignant evolution of tumor tissue—including aberrant proliferation, angiogenesis, invasion, metastasis, and the remodeling of the tumor microenvironment (TME). First, as a co-factor for key enzymes and a signaling molecule, Cu^2+^ directly promotes rapid tumor cell proliferation and metabolic reprogramming by activating pathways such as MAPK and PI3K/Akt while stabilizing HIF-1α, establishing a state of “copper addiction.” Second, copper drives the HIF-1α/VEGF axis and activates lysyl oxidase (LOX), thereby promoting neovascularization and extracellular matrix remodeling, which supplies nutrients to the tumor and paves the way for metastasis. Furthermore, copper enhances tumor cell migration and invasive capacity by inducing epithelial–mesenchymal transition (EMT) and upregulating LOX, and contributes to the formation of pre-metastatic niches. Concurrently, disrupted copper homeostasis also fosters therapeutic resistance: elevated Cu^2+^ levels enhance antioxidant defenses, while upregulation of copper-transporting ATPases such as ATP7A/B accelerates the efflux of chemotherapeutic agents, enabling tumor cells to evade conventional therapies as well as novel cell death modalities like cuproptosis. These processes are deeply intertwined with TME. Copper ions can suppress effector immune cell functions and polarize immune cells toward immunosuppressive phenotypes, while simultaneously influencing stromal cells via intercellular signaling, collectively fostering a permissive micro-ecosystem that supports tumor growth, metastasis, and immune evasion. Therefore, copper dyshomeostasis acts as a core driver throughout the entire continuum of tumor malignancy [98,99,100].

FDX1 directly regulates the acylation process of TCA cycle enzymes, and so its expression level is the primary indicator for evaluating the cuproptosis sensitivity of tumor cells. The expression levels of acylating enzymes such as DLAT themselves and their aggregation states within cells serve as direct evidence of whether cuproptosis occurs. For instance, in liver cancer and colon cancer, the upregulation of DLAT is closely related to the differentiation and proliferation activities of tumor cells. In addition to these core executors, tumor cells maintain copper homeostasis by precisely regulating the copper “ingestion–transport–excretion” network, thereby evading cuproptosis. The key molecules in this network have become another important type of biomarker for predicting cuproptosis sensitivity and drug resistance. The high expression of CTR1 in breast cancer and thyroid cancer may indicate the increased dependence of tumor cells on copper and make them more vulnerable to copper ion carriers or high-copper environments. That is to say, it marks a type of tumor subtype sensitive to cuproptosis induction therapy [101,102]. On the contrary, the copper excrete proteins ATP7A and ATP7B are the main lines of defense cells possess against copper toxicity, and their high expression is the core mechanism of cuproptosis and drug resistance in tumors. Research has found that in breast cancer, the activation of the C5a/C5aR pathway upregulates ATP7B through Wnt/β-catenin signaling, promoting copper excretion and leading to the resistance of cancer cells to cuproptosis inducers. Similarly, in cancer stem cells, the Wnt/β-catenin pathway can directly promote the transcription of ATP7B, endowing it with cuproptosis resistance. Therefore, the expression level of ATP7A/B is a negative biomarker of cuproptosis, and its increase indicates that the related therapy may fail. What is more complex is that multiple classic oncogenic or tumor suppressor signaling pathways have been found to profoundly influence the threshold of cuproptosis by regulating the above-mentioned core and steady-state molecules. In colorectal cancer and triple-negative breast cancer, the activation of the key survival pathway PI3K/AKT/mTOR has been demonstrated to phosphorylate and inhibit the activity of FDX1, thereby directly turning off the cuproptosis switch. Its high phosphorylation level is a powerful negative marker for predicting cuproptosis resistance [103,104]. HIF-1α is activated in the hypoxic microenvironment of solid tumors and can promote cuproptosis by upregulating CTR1. Its activity can be used as an environmental marker for predicting the possibility of cuproptosis. In lung adenocarcinoma, the activation of the SPI1/CDKN2A/p53 axis can enhance the sensitivity of cancer cells to cuproptosis. In hepatocellular carcinoma, SEC14L3 positively regulates cuproptosis and inhibits tumor growth by activating the ERK signal and promoting the upregulation of FDX1 expression by the transcription factor YY1.

Cuproptosis is not an isolated cellular event; it can profoundly reshape the tumor immune microenvironment by releasing damage-associated molecular patterns (DAMPs). Studies have shown that cuproptosis leads to the release of mitochondrial DNA (mtDNA) into the cytoplasm, activating the cGAS-STING innate immune pathway, which in turn induces IFN production and immunogenic cell death (ICD), recruiting and activating cytotoxic T lymphocytes [105,106]. Therefore, the activation status of the STING pathway, the level of T cell infiltration, and the release of ICD-related molecules HMGB1 and ATP can serve as biomarkers for the immunogenic effects of cuproptosis. However, cuproptosis resistance or specific cuproptosis-related genes are usually associated with an immunosuppressive microenvironment, manifested as reduced CD8+ T cell infiltration and upregulation of PD-L1, suggesting that these immune features can serve as comprehensive markers for evaluating the overall biological effects of cuproptosis [107].

We note that the current understanding of the aforementioned vast biomarker network is largely attributed to the large-scale bioinformatic mining of public databases such as the Cancer Genome Atlas (TCGA) and the Comprehensive Gene Expression (GEO). This method has revolutionary advantages. It enables us to systematically screen molecules significantly associated with cuproptosis and clinical prognosis in a pan-cancer type in an unbiased and high-throughput manner. However, its inherent limitations must also be guarded against. Bioinformatics mainly reveals statistical correlations rather than causal relationships. A molecule’s correlation with prognosis does not necessarily prove that it functionally participates in copper mortality. The mRNA expression levels in the database may not fully reflect the true functional states of proteins, such as their abundance, post-translational modifications, and subcellular localization (Table 2).

### 6.2. Neurological Diseases

In neurological diseases, research on cuproptosis has been widely applied to various conditions such as stroke, Alzheimer’s disease, amyotrophic lateral sclerosis, Parkinson’s disease, major depressive disorder, and epilepsy. In stroke research, six key cuproptosis-related genes, namely NLRP3, NFE2L2, ATP7A, LIPT1, GLS, and MTF1, were identified by integrating multiple transcriptome datasets. These genes promote acylation metabolism and mediate downstream oxidative stress and inflammatory responses by sensing copper ion stress and the outward transport and excretion of copper. It is suggested that it plays a core role in the regulation of cuproptosis in ischemic stroke [129,130]. Research on Alzheimer’s disease (AD) focuses on genes such as IFI30, PLA1A, ALOX5AP and A4GALT, which are involved in antigen processing and presentation, phospholipid metabolism, inflammatory response, and sphingolipid synthesis, and can promote Aβ levels and β -secretase activity. It further reveals that cuproptosis may play a role in neurodegeneration through both immune and metabolic pathways [131]. It is noteworthy that, beyond the dysregulation of cuproptosis-related genes, non-ceruloplasmin copper may also serve as a key indicator of cuproptosis in AD. Multiple clinical studies have demonstrated elevated levels of “free copper” in both the blood and brains of AD patients, and an imbalance of non-ceruloplasmin copper has been observed, even at the prodromal stage of AD. Importantly, zinc supplementation has been shown to effectively reduce non-ceruloplasmin copper in AD patients [132,133,134]. The increase in “free copper” is often associated with functional or genetic variations in the copper-transporting protein ATP7B. A clinical study identified four specific single-nucleotide polymorphisms in the ATP7B gene among AD patients, supporting the presence of genetic alterations in this pathway. ATP7B, primarily expressed in hepatocytes, functions as an efflux transporter responsible for loading copper onto ceruloplasmin and facilitating its biliary excretion. Impaired ATP7B activity thus leads to the accumulation of “free copper” in the systemic circulation. This bioactive copper fraction can cross the blood–brain barrier, accumulate in neuronal mitochondria, and disrupt mitochondrial energy metabolism. As a central site for cellular energy production and copper utilization, mitochondria are vulnerable to copper overload, which may trigger cuproptosis—a process involving direct copper-mediated lipoylation of mitochondrial enzymes, resulting in metabolic collapse and cell death. Therefore, measuring serum non-ceruloplasmin copper not only provides a robust tool for identifying this distinct AD subtype, but also offers a potential biomarker for guiding precision interventions targeting copper homeostasis. Furthermore, the formation of pathological hallmarks in other neurodegenerative diseases, such as Parkinson’s disease, amyotrophic lateral sclerosis, and Huntington’s disease, is closely linked to cuproptosis. In Parkinson’s disease, Cu^2+^ engages in aberrant interactions with α-synuclein and exacerbates the metabolic vulnerability of dopaminergic neurons. In amyotrophic lateral sclerosis and Huntington’s disease, copper-mediated oxidative damage and mitochondrial dysfunction synergize with TDP-43 pathology and the toxicity of mutant huntingtin protein (mHTT), respectively, collectively driving the progressive loss of motor neurons and striatal neurons. Thus, cuproptosis is not an isolated event but rather a shared pathological pathway that connects metal homeostasis imbalance, mitochondrial energy failure, oxidative stress, and disease-specific protein toxicity. In amyotrophic lateral sclerosis, BAP1, SMG1, and BCLAF1 are recognized. Although these genes do not directly participate in the regulation of cuproptosis, they can indirectly affect the expression of genes related to cuproptosis by regulating DNA damage response and apoptosis-related factors, thereby playing a regulatory role [135]. Research on Parkinson’s disease has focused on three key genes: SLC18A2, SLC6A3, and SV2C. These cuproptosis markers are mainly transporters of vesicle monoamine neurotransmitters and glycoproteins on synaptic vesicles, which regulate the homeostasis and vesicle function of dopamine (DA), norepinephrine (NE), and serotonin (5-HT). It affects the sensitivity of nerve cells to copper ion stress, thereby indirectly participating in the cell type-specific regulation of cuproptosis [136]. In major depressive disorder, genes such as OSBPL8 and VBP1 have been incorporated into the predictive model. These two genes are more like “regulators” of cellular homeostasis. They are not direct targets or executors of cuproptosis, but the lipid metabolism and protein homeostasis they maintain form the basis for cells to cope with copper ion overload [137]. Research on epilepsy has emphasized the phasic changes of LIPT1 and FDX1 in their infiltration with macrophages and T cells during acute and chronic disease courses. LIPT1 installs lipoic acid into the subunits of the pyruvate dehydrogenase complex and the α-ketoglutarate dehydrogenase complex to maintain the stability of energy metabolism. However, the high expression of LIPT1 and FDX1 in epilepsy leads to disorders in the TCA cycle and copper transport, resulting in insufficient energy synthesis and accumulation of lactic acid and pyruvate, promoting cuproptosis and inducing neuroinflammation in epilepsy [138].

### 6.3. Cardiovascular Diseases

The myocardium and blood vessels are the tissues most sensitive to changes in mitochondrial function. Stressors such as hypertension and ischemia first trigger oxidative stress, leading to the excessive generation of reactive oxygen species. Reactive oxygen species attack polyunsaturated fatty acids on the cell membrane, causing lipid peroxidation and generating toxic aldehyde products. This leads to the abnormal accumulation of free Cu^2+^ with REDOX activity in mitochondria, which directly bind to acylated proteins in the tricarboxylic acid cycle, resulting in mitochondrial metabolic dysfunction and triggering cuproptosis [139]. Ultimately, the massive death of myocardial cells and vascular cells directly leads to myocardial contractility failure and vascular dysfunction, exacerbating the progression of diseases such as heart failure and atherosclerosis. In the cardiovascular system, cuproptosis is involved in myocardial infarction, dilated cardiomyopathy, primary cardiomyopathy, atherosclerosis, and myocardial ischemia–reperfusion injury. In acute myocardial infarction (AMI), the expression of six genes is significantly abnormal: LIAS, LIPT1, DLAT, PDHB, MTF1, and GLS. MTF1 acts as an upstream regulator to sense copper ion concentration, while LIAS and LIPT1 are responsible for catalyzing protein acylation modification. DLAT and PDHB, as acylated proteins in the tricarboxylic acid cycle, directly bind to copper ions and undergo toxic aggregation. GLS provides the necessary substrate α -ketoglutaric acid for this process by hydrolyzing glutamine. They together constitute the core metabolic pathway of cuproptosis [140]. The initiating factors of dilated cardiomyopathy are genetic defects or acquired injuries, which trigger myocardial cell death and dysfunction, thereby activating the neuroendocrine system and pro-fibrotic pathways, leading to progressive cardiac dilation and weakened contractility [141]. Six characteristic genes, including SEPTIN1, CLEC11A, ISG15, P3H3, SDSL and INKA1, were screened out from the samples of patients with dilated cardiomyopathy, and ceRNA and drug regulatory networks were constructed, providing a new approach for the molecular typing and treatment of DCM [142]. In addition, studies on primary cardiomyopathy have found that MAP2K1, FDX1, and CTR1 are common cuproptosis marker genes for the three types of primary cardiomyopathy. CTR1 mediates the uptake of Cu^2+^ by cells, while FDX1 reduces Cu^2+^ to the more toxic Cu^+^ and directly drives the oligomerization of acylated proteins in cardiomyocytes. MAP2K1 may affect the stress activity of cells in response to cuproptosis by regulating downstream signaling pathways such as ERK [143]. Atherosclerosis studies have for the first time revealed abnormal expression of FDX1, CTR1 and GLS in plaque tissues, suggesting that these genes can affect the transmembrane transport level of copper and mediate the occurrence of AS [144]. Studies on myocardial ischemia–reperfusion injury (MI/RI) have emphasized that the downregulation of DLAT, PDHB, and PDH1 is a potential biomarker for MI/RI, among which PDHB has a key diagnostic value [145].

### 6.4. Digestive System Diseases

The accumulation of copper ions within cells directly inhibits lipoacylated proteins (DLAT, DLST) in the TCA cycle, leading to abnormal aggregation of lipoacylated proteins, obstruction of the mitochondrial respiratory chain, and the production of a large amount of reactive oxygen species, thereby inducing intense oxidative stress. Subsequently, this mitochondrial stress and cell death will release damage-related molecular patterns including mitochondrial DNA and ATP, thereby activating the NLRP3 inflammasome signaling pathway and promoting the release of inflammatory factors IL-1β and IL-18. This not only directly amplifies the inflammatory response of local tissues and disrupts the integrity of the intestinal mucosal barrier, but it also further increases the copper ion flux and oxidative stress within the cells, ultimately continuously driving the pathological progression and tissue damage of enteritis or peptic ulcers [146,147]. In digestive system diseases, research on cuproptosis has focused on inflammatory bowel diseases, including Crohn’s disease (CD) and ulcerative colitis. In the study of Crohn’s disease, 25 differentially expressed cuproptosis-related genes, including ABCB6, FDX1, GLS, LIAS, MT1M, and PDHA1, were identified. Most of them were negatively correlated with immune cell infiltration. Further cluster analysis revealed two subgroups with different immune and metabolic characteristics. It is suggested that cuproptosis jointly promotes the progression of CD through immune responses and metabolic disorders [148]. In the research on inflammatory bowel disease, it was found that genes such as PDHA1, DBT, DLAT, and LIAS are co-differentially expressed in Crohn’s disease, ulcerative colitis, celiac disease, and IBD-related cancers, with the main pathway concentrated in mitochondrial respiratory function. This further confirmed that PDHA1, DLAT, and LIAS are the key cuproptosis markers mediating the occurrence of IBD [149]. Research on ulcerative colitis has identified six hub genes, including ATOX1, SUMF1, MT1G, ATP7B, FDX1 and LIAS. Among them, ATOX1, SUMF1, MT1G and ATP7B, as negative regulators of copper homeostasis, inhibit cuproptosis by mediating the partner transport and excretion of copper. FDX1 and LIAS, as core positive regulatory factors, perform cuproptosis by reducing copper ions to drive the oligomerization of acylated proteins and catalyze key acylation modifications [150].

### 6.5. Bone and Muscle Diseases

Abnormally elevated Cu penetrates into the mitochondria of cells, leading to toxic oligomerization and accumulation, directly inducing the death of osteoblasts, chondrocytes, nucleus pulposus cells and muscle cells, resulting in structural damage such as muscle fiber atrophy, subchondral bone sclerosis, intervertebral disc matrix degradation, and insufficient bone formation. Meanwhile, the DAMP released by dead cells can continuously activate IL-1β and TNF-α, intensifying synovial inflammation and cartilage erosion, and hindering tissue repair and promoting osteoclasts/protein breakdown in osteoporosis and sarcopenia [151]. Among the diseases of this system, research on cuproptosis covers a variety of conditions such as sarcopenia, intervertebral disc degeneration, osteoarthritis, rheumatoid arthritis, spinal cord injury and osteoporosis. In sarcopenia, the AUC value of the diagnostic model constructed by 11 cuproptosis-related genes was as high as 0.856. The study also found that Treg cells decreased in the muscle tissue of the elderly, while dendritic cells and mast cells increased, suggesting that cuproptosis-mediated immune cell dysfunction plays a key role in muscle aging [152,153]. The study on intervertebral disc degeneration screened out eight key regulatory factors such as LIPT1, ATP7A, and MTF1. Functional enrichment indicated that they were closely related to the tricarboxylic acid cycle, suggesting that energy metabolism disorder is the core mechanism of cuproptosis in intervertebral disc degeneration [154]. In osteoarthritis, predictive models constructed by seven genes such as DBT and LIPT1, combined with cluster analysis, identified two subtypes with different expression profiles of inflammatory factors, suggesting that cuproptosis is involved in disease progression in OA by regulating inflammatory pathways such as IL-1β and IL-17 [155]. Rheumatoid arthritis has found that FAM96A and CGRRF1 are significantly highly expressed in the patient’s serum. FAM96A negatively regulates the assembly of iron–sulfur clusters by binding to and inhibiting the key protein CIA2A for iron–sulfur cluster biosynthesis, while CGRRF1, as a novel copper partner protein, jointly sense and transmit copper stress signals. Thus, at the upstream regulatory level of cuproptosis, this cell death process can be precisely regulated, which provides a new target for the immune-metabolic intervention of RA [156]. Spinal cord injury research emphasizes that DLD is positively correlated with M2-type macrophage infiltration, and NT3-loaded chitosan can promote neurological function recovery by inhibiting the expression of cuproptosis related genes, suggesting its therapeutic potential [157]. In osteoporosis, the predictive model composed of five genes including EVA1B and RTCB has shown stable performance in external validation. EVA1B, RTCB, HEXB, SLC25A11 and TMEM126B regulate autophagy, tRNA stress repair, lysosomal function and mitochondrial metabolism. In cuproptosis, they jointly form a complex monitoring and defense network, indirectly affecting the cells’ tolerance to copper ion toxic stress [158].

### 6.6. Immune System Diseases

Among immune system diseases, sepsis, Sjogren’s syndrome, systemic lupus erythematosus, tuberculosis, Behcet’s disease, and allergic rhinitis have all been confirmed to be closely related to copper mortality. Sepsis studies have found that the expression of most cuproptosis-related genes is upregulated. The diagnostic models constructed by LIAS and PDHB demonstrate superior discriminative ability and show tissue-specific expression patterns in multiple organs, suggesting that they have mechanism heterogeneity in multi-organ injury of sepsis [159]. In Sjogren’s syndrome, EED, CBL and NFU1 are lowly expressed in the patient’s serum, suggesting that cuproptosis plays a regulatory role in the immune disorder of pSS [160]. Systemic lupus erythematosus research emphasizes the downregulation of LIAS and the upregulation of CDKN2A. The correlation between LIAS and interferon pathways and cellular communication networks reveals a novel mechanism of cuproptosis in the immune dysregulation of SLE [161]. Tuberculosis research has revealed 11 differentially expressed cuproptosis genes, among which NFE2L2 and NLRP3 are upregulated, while LIAS and LIPT1 are downregulated, suggesting that Mycobacterium tuberculosis infection may affect the host immune response by regulating copper homeostasis [162]. Six hub genes such as ANKRD9 and COX11 have been identified in Cet’s disease. The immune activity of their high-expression subtypes is enhanced, emphasizing the immunomodulatory role of cuproptosis in vasculitic lesions [163]. Allergic rhinitis research has identified four characteristic genes: MRPS30, CLPX, MRPL13, and MRPL53. Among them, MRPS30, MRPL13, and MRPL53 mainly regulate the synthesis of mitochondrial-encoded proteins, and CLPX promotes the misfolding and degradation of mitochondrial proteins under copper stress. This further affects the assembly and function of mitochondrial respiratory chain complexes and regulates the sensitivity of cells to copper ion toxicity [164].

### 6.7. Endocrine and Metabolic System Diseases

Cuproptosis plays a significant role in the pathological formation of endocrine and metabolic diseases by disrupting cellular energy metabolism and triggering inflammatory cascade reactions. In the liver, the imbalance of copper ion metabolism leads to the abnormal accumulation of copper within cells, causing the high expression of ROS and low expression of SOD and directly inducing steatosis, ballooning, and even the death of hepatocytes [165]. In diabetes, oxidative stress and inflammatory responses mediated by cuproptosis not only exacerbate insulin resistance in peripheral tissues but also may directly damage the function of pancreatic β cells, induce their apoptosis, and lead to insulin secretion defects, thereby participating in the vicious cycle of the occurrence, development and metabolic disorders of diabetes [166]. It is currently known that the presence of cuproptosis in this system, non-alcoholic fatty liver disease, alcoholic hepatitis, and diabetes are the research focuses. Three hub genes, NFE2L2, DLD and POLD1, were screened out for non-alcoholic fatty liver disease through machine learning. Among them, DLD and PDHB were stably upregulated in multiple datasets, suggesting that cuproptosis plays a core role in the metabolic disorder of NAFLD [167]. Studies on alcoholic hepatitis have found that M1 macrophage infiltration is positively correlated with ferroptosis and copper-ptosis scores. Genes such as ALDOA and COL3A1 are identified as common biomarkers for the three, revealing their hub role in the AH immune metabolic network [168]. Diabetes research focuses on the severe limb ischemia model of diabetes, and it has been found that copper overload and cuproptosis-related proteins such as CTR1 and ATP7A are abnormally expressed. The copper chelating agent ammonium tetrathiopolybate can significantly alleviate cell death and tissue damage, providing a new treatment idea for vascular complications of diabetes [169].

### 6.8. Respiratory Diseases

Among respiratory diseases, copper mortality studies cover idiopathic pulmonary fibrosis, asthma, pneumonia, influenza and pulmonary arterial hypertension. In idiopathic pulmonary fibrosis, five genes (AKAP9, ANK3, C6orf106, LYRM7 and MBNL1) have been established as prognostic markers, suggesting that the cuproptosis score can be used as a risk stratification tool for IPF patients [170]. AKAP9, as an A-kinase anchor protein, and ANK3-encoded anchor protein B are abnormally expressed in epithelial cells and fibroblasts, intensifying the differentiation and proliferation of fibroblasts into myofibroblasts, weakening the epithelial barrier function, and promoting the occurrence of epithelial–mesenchymal transition. LYRM7 is an important cofactor for the assembly of iron–sulfur clusters; functional defects directly lead to the assembly failure and functional impairment of mitochondrial respiratory chain complex III. Meanwhile, MBNL1 is a key RNA-binding protein; its expression imbalance will cause errors in the selective splicing of a large number of precursor MRNAs. This directly promotes the continuous activation of myofibroblasts and the excessive production of collagen. Asthma research has identified five characteristic genes (RIM25, DYSF, NCF4, ABTB1, CXCR1). The dysregulation of these genes jointly disrupts airway homeostasis, promotes inflammatory cell infiltration and persistent inflammation, and ultimately leads to typical pathological changes of asthma such as airway hyperresponsiveness, excessive mucus secretion and airway remodeling [171]. Abnormal function of NCF4 can impair the reactive oxygen species production of immune cells. Instead, it may cause chronic inflammation due to the inability to effectively eliminate pathogens and apoptotic cells, and even promote the development of neutrophilic asthma. As a scaffold protein, the reduction in ABTB1’s function will result in the loss of negative regulation of inflammatory pathways such as NF-κB through the ubiquitination pathway, leading to excessive activation of inflammatory signals. CXCR1 directly guides neutrophils to recruit and activate in the airway by mediating chemokines such as IL-8, becoming the key driver of neutrophilic inflammation in severe asthma. Pneumonia research has found that nine cuproptosis-related genes are co-expressed in severe community-acquired pneumonia. They are all associated with immune cell infiltration, suggesting that they have a broad role in the immunopathology of pneumonia [172]. Influenza studies have identified two subtypes of copper-death associated with severe influenza. Among them, subtype 1 is accompanied by a decline in adaptive immunity and activation of neutrophils, suggesting that copper-death may be related to the severity of influenza and immune escape. The research on pulmonary arterial hypertension takes AHR, FAS and FGF2 as key diagnostic markers [173]. Excessive activation of AHR can inhibit the key antiviral type I interferon response and weaken the virus clearance ability. Regarding Fas-mediated apoptosis, while eliminating infected cells, excessive activation can lead to the massive death of lymphocytes and lung epithelium, exacerbating immunosuppression and tissue damage. If the tissue repair signal driven by FGF2 is abnormally enhanced, although it is helpful for epithelial recovery, it may promote excessive proliferation of fibroblasts and extracellular matrix deposition, potentially initiating the process of pulmonary fibrosis.

### 6.9. Other Systemic Diseases

Other systemic diseases include gynecological diseases, skin diseases, kidney diseases, and oral diseases. In endometriosis, PDHA1 is downregulated and inhibits cuproptosis through the PI3K-Akt-mTOR pathway, suggesting its association with the high proliferation characteristics of EM [174]. Five key genes such as COL5A1 and IL18BP were identified for polycystic ovary syndrome, and the constructed predictive model provided a basis for the molecular typing of PCOS [175]. Psoriasis research has found that MTF1, ATP7B, and CTR1 are significantly expressed, and their functional enrichment suggests that they are closely related to acetyl-CoA metabolism and mitochondrial function [176]. In terms of nephropathy, four key markers such as LIPA and LIPT1 were identified in renal ischemia–reperfusion injury, and the diagnostic performance of the model constructed by machine learning was excellent [177]. In diabetic nephropathy, FSTL1, CX3CR1, and AGR2 are significantly correlated with renal function indicators. As a pro-inflammatory and pro-fibrotic mediator, FSTL1 directly stimulates the activation of glomerular mesangial cells and renal tubular epithelial cells, enhances the TGF-β signaling pathway, and accelerates the abnormal deposition of extracellular matrix. CX3CR1 promotes local inflammatory infiltration and amplifies immune damage by mediating the specific migration and retention of monocytes/macrophages to renal tissue, thereby intensifying the production of proteinuria. However, AGR2 is abnormally expressed under endoplasmic reticulum stress conditions, leading to the apoptosis of renal tubular epithelial cells and weakening the inherent protective mechanism of the kidneys [178]. Research on chronic renal fibrosis emphasizes that genes such as Bcl2a1b and Clec4n are closely related to inflammation and cuproptosis processes [179]. Periodontitis research has found through single-cell sequencing that GLS are widely expressed in immune cells, suggesting that they play a core role in the regulation of the periodontal immune microenvironment [180]. Highly expressed GLS can catalyze the decomposition of glutamine to produce a large amount of glutamic acid and tricarboxylic acid cycle intermediate products. This process promotes IL-1β and TNF-α; also, the accumulated glutamic acid can directly act as a signaling molecule to activate channels such as NMDA receptors, intensifying intracellular calcium overload and oxidative stress (Table 3).

## 7. Targeted Therapy for Cuproptosis

### 7.1. Copper Chelator

Copper chelators have demonstrated therapeutic potential across various disease models by inhibiting the copper-induced cell death process through the reduction of intracellular copper ion concentrations. Notably, penicillamine (PA), D-penicillamine (DP), tetramolybdate (TTM), ammonium tetramolybdate (ATTM), and triethylenetetramine (TETA) have all been documented in the literature. PA, a well-established copper chelating agent, has shown significant efficacy in the treatment of inflammatory bowel disease and Wilson’s disease. Studies indicate that PA mitigates intestinal barrier damage by lowering the abnormally high copper levels in intestinal tissues, inhibiting the oligomerization of dihydrothiocarcinamide acetyltransferase (DLAT), and concurrently preserving the expression of ferridoxin 1 (FDX1) and lipoate synthase (LIAS) [182]. PA has also shown protective effects in models of doxorubicin-induced cardiotoxicity. It alleviated myocardial fibrosis and improved cardiac function by regulating the expression of SOD, glutathione peroxidase (GPX), malondialdehyde (MDA), and cuproptosis-related proteins, including FDX1, LIAS, CTR1, and ATP7A [183]. DP’s research further validated the efficacy of these drugs in regulating copper homeostasis. Additionally, their long-term application in rheumatoid arthritis and Wilson’s disease offers a safety reference for tumor treatment [184]. Furthermore, early clinical evidence highlights the distinctive therapeutic potential of D-penicillamine (DP) in AD. This is primarily reflected in the observation that erythrocyte copper–zinc superoxide dismutase (Cu,Zn-SOD) activity is significantly elevated in AD patients even at early disease stages. This abnormality in a peripheral copper-dependent enzyme indicates systemic copper dysmetabolism. More importantly, the same study confirmed that a 24-week treatment with DP effectively reduced Cu,Zn-SOD activity in AD patients to levels below those of healthy controls [185]. This finding not only establishes Cu,Zn-SOD activity as an early peripheral biomarker for AD, but crucially demonstrates that copper dyshomeostasis in AD is pharmacologically modifiable and can be monitored via peripheral biomarkers—thereby providing preliminary clinical validation for the translational application of copper chelation therapy. Additionally, recent studies on zinc therapy in mild cognitive impairment (MCI) and AD further support the clinical feasibility of modulating copper homeostasis. Zinc induces intestinal metallothionein synthesis, inhibits dietary copper absorption, and thereby effectively lowers serum levels of toxic non-ceruloplasmin copper. Together, these interventional studies suggest that regulating copper homeostasis represents a biologically rational and clinically responsive treatment strategy for the “copper-overload” subtype of AD. TM and its derivative ATTM exhibit a broader spectrum of action. In the pulmonary fibrosis model, it not only reduces the copper level in lung tissue, but also reverses the epithelial–mesenchymal transition and fibroblast–myofibroblast transition by inhibiting the transforming growth factor -β (TGF-β) signaling pathway, and alleviates collagen deposition [186]. In the periodontitis, TM mitigates the inflammatory response by restoring the autophagy–lysosomal function of macrophages, normalizing the acidic environment of lysosomes, and decreasing the secretion of cathepsin B [187]. Notably, ATTM has exhibited a regulatory effect on YTHDF1-dependent m6A methylation in lung adenocarcinoma research. This finding connects copper homeostasis to epigenetic regulation and broadens the understanding of the mechanisms by which copper chelators operate [188]. TETA, another copper chelating agent used in clinical settings, has limited related studies to date. However, its successful application in treating Wilson’s disease indicates that it may possess similar potential for regulating copper-induced cell death in tumors [189]. These findings collectively indicate that the role of copper chelating agents extends beyond the mere binding of metal ions; it encompasses the precise regulation of various signaling pathways.

### 7.2. Copper Transport Carrier Agent

Copper ion carriers exhibit distinct advantages in tumor treatment by elevating intracellular copper ion concentrations and specifically inducing copper-mediated cell death. Disulfiram (DSF) and elesclomol serve as prominent examples, while other copper ion complexes also demonstrate promising potential. Research on disulfiram has underscored its considerable value in combination therapy. In non-small-cell lung cancer, the disulfide metabolite diethyldithiocarbamate forms a toxic complex, CuET, with copper ions. This complex not only enhances the expression of FDX1 and promotes the aggregation of DLAT, but also induces immunogenic cell death by inhibiting proteasome function. The combined application of disulfide and PD-L1 inhibitors resulted in a synergistic effect, markedly increasing the cuproptosis effect through the upregulation of reactive oxygen species levels and the downregulation of HIF-1α expression [190]. In malaria-related acute lung injury, disulfide exacerbates the inflammatory response by promoting M1 polarization of macrophages and upregulating the expression of FDX1 and CTR1, along with increasing copper levels. This environmental dependence indicates the necessity for careful selection of therapeutic indications [191]. Elesclomol is recognized for its exceptional ability to target mitochondria. In the context of pancreatic ductal adenocarcinoma, the concurrent administration of Elesclomol and copper chloride can elevate intracellular copper ion levels by 2 to 4 times. This combination specifically reduces iron–sulfur cluster proteins and leads to the accumulation of DLAT in mitochondria, thereby selectively eradicating cancer stem cells [192]. Notably, the typical features of copper-induced cell death, as observed in oyster blood cells following Elesclomol treatment, include a decrease in mitochondrial membrane potential, destruction of crest structures, and alterations in metabolite levels. These findings provide evidence for the evolutionary conservation of cuproptosis [193]. In acute myeloid leukemia, Elesclomol-Cu demonstrates concentration-dependent inhibitory effects on proliferation, which are associated with increased levels of reactive oxygen species and reduced glutathione. This observation supports the need for dose optimization in its clinical application [194]. Notably, typical features of copper-induced cell death, such as diminished mitochondrial membrane potential, disruption of crest structures, and alterations in metabolite levels, were observed in oyster blood cells. These findings provide evidence for the evolutionary conservation of copper-induced cell death. In acute myeloid leukemia, Elesclomol-Cu demonstrates concentration-dependent inhibitory effects on proliferation, which are associated with increased levels of reactive oxygen species and depletion of glutathione. This observation establishes a foundation for optimizing dosage in clinical applications.

### 7.3. Natural Products

Research on the regulation of copper-induced cell death by natural products has progressed from initial observational studies to a focus on molecular mechanisms and the development of innovative therapeutic strategies. These products exhibit significant therapeutic potential across various domains, including cancer, neurological disorders, and cardiovascular and metabolic diseases, by modulating the cuproptosis pathway. The mechanisms of action can be primarily categorized into several groups, revealing intriguing regulatory differences across diverse disease contexts.

In the realm of tumor treatment, a fundamental strategy involving natural products is the induction of cuproptosis to eliminate cancer cells. However, the specific mechanisms underlying this process are diverse, reflecting the complexity of tumor metabolism and the variety of targeting strategies employed. Certain compounds, such as curcumin, function as “copper homeostasis disruptors” [195]. These agents not only enhance the expression of copper uptake proteins CTR1 and CTR1 but also inhibit the copper efflux proteins ATP7A and ATP7B. This dual action results in copper ions entering cancer cells without being expelled, ultimately surpassing a critical threshold to trigger cuproptosis. Epigallocatechin Gallate reduces copper excretion by inhibiting the MTF1/ATP7B axis, increases copper accumulation in liver cancer cells, and thereby enhances their sensitivity to cuproptosis [196]. Complanatoside A induces cuproptosis in prostate cancer cells by downregulating the copper chaperone protein ATOX1, leading to copper ion accumulation and mitochondrial dysfunction [197]. Taxifolin triggers cuproptosis in liver cancer cells by upregulating copper intake mediated by the copper import protein CTR1 [198]. Cinobufagin promotes intracellular copper accumulation and oxidative stress by regulating copper transporters (upregulating CTR1/2 and downregulating ATP7A/B), thereby enhancing cuproptosis in liver cancer cells [199]. This indicates that targeted copper transport systems may exhibit greater specificity than the direct application of exogenous copper ion carriers. Additionally, compounds such as baicalein, triptolide and plumbagin influence copper homeostasis indirectly by disrupting the upstream signaling pathways, PI3K/Akt, XIAP/COMMD1 or DNMT1/miR-302a-3p. This finding highlights the potential role of epigenetic regulation in copper-induced cell death and offers new insights for combination therapy [200,201]. Eupalinolide B disrupts the copper homeostasis of pancreatic cancer cells, enhances the accumulation of mitochondrial reactive oxygen species, and collaborates with the copper ion carrier elesclomol to induce cuproptosis [202]. Chlorophyllin enhances the sensitivity of pancreatic cancer cells to gemcitabine by depleting glutathione, increasing reactive oxygen species and promoting FDX1-dependent DLAT oligomerization [203]. The research conducted by quercetin and dihydroartemisinin has uncovered a more intricate mechanism. By reprogramming the metabolic state of tumor cells, these compounds enhance mitochondrial respiration or inhibit glycerophospholipid metabolism, thereby increasing the sensitivity of cancer cells to copper-induced death. This finding underscores the sophistication of the “metabolic sensitization” strategy, which manifests as an alteration of inherent vulnerabilities rather than a direct cytotoxic effect [204,205]. The natural product pochonin D, derived from microorganisms, has been recognized as a novel carrier of copper ions. It covalently binds to the Cys173 site of the PRDX1 protein, inhibiting its peroxidase activity and inducing a form of cell death resembling copper-induced death, independent of the acylated TCA cycle enzyme, in triple-negative breast cancer [206]. Tan IIA can promote the expression of FDX1 and the formation of cuproptosis in tumor cells through the increasing FDX1 m6A modification mediated by METTL3/METTL14, exerting an anti-bladder cancer effect. Furthermore, glycyrrhizic acid, tremella fuciformis polysaccharide, shiborin and aloe emodin, when combined with nanotechnology, facilitate the construction of an intelligent drug delivery system. This system achieves synergy between cuproptosis, photodynamic therapy, and immunotherapy, marking a significant advancement in the future treatment of tumors [207,208].

In contrast to its “pro-death” effect in tumors, the role of natural products in protecting against non-cancerous diseases has shifted to the inhibition of copper-induced cell death, thereby safeguarding normal cells. This bidirectional regulatory capacity underscores the pathological significance of the cuproptosis pathway and the context-dependent nature of intervention strategies. In cardiovascular diseases, paeoniflorin and phillygenin mitigate cuproptosis in myocardial infarction and ischemia–reperfusion injury by downregulating FDX1/DLAT or promoting the degradation of CTR1, thereby enhancing cardiac function [209,210]. This finding strongly indicates that cuproptosis represents a novel and targetable mechanism in myocardial injury. Trilobatin directly binds to FDX1, inhibiting doxorubicin-induced cuproptosis in cardiomyocytes, reducing cardiotoxicity, and improving cardiac function [211]. In contrast to its “pro-death” role in tumors, the primary function of natural products in the context of non-cancerous diseases has shifted toward inhibiting cuproptosis to protect normal cells. This bidirectional regulatory capacity underscores the pathological significance of the cuproptosis pathway and the situational dependence of intervention strategies. Research on artesunate in the nervous system is particularly noteworthy. Artesunate chelates excessive copper ions by upregulating metallothionein MT2A. Both compounds have demonstrated neuroprotective effects in models of Parkinson’s disease [212,213]. Icaritin can directly bind to and downregulate the key protein FDX1 of cuproptosis, alleviate the mitochondrial damage and cuproptosis of neurons induced by copper ion carriers, and has neuroprotective potential [214]. These findings confirm the role of cuproptosis in neurodegenerative diseases and present two distinct neuroprotective strategies: “upstream blocking” and “source clearance.” Likewise, in cases of liver and kidney injuries, traditional Chinese medicines Qiju Dihuang pills and quercetin inhibit cuproptosis by modulating m6A modification, which reduces CTR1 levels and decreases free copper ions, thereby providing neuroprotective effects [215,216]. Diallyl trisulfides specifically activates cuproptosis in hepatic stellate cells in liver fibrosis models by inducing RAB18 phase separation and promoting the formation of mitochondrial-associated membrane structures, while protecting normal liver cells [217]. The consistent protective effect observed across various organs has established a theoretical foundation for cuproptosis inhibitors as a novel category of organ protective agents. In the burgeoning field of anti-inflammation and immune regulation, natural products have exhibited distinct bidirectional potential in modulating copper mortality. Research on Syringaresinol-4-O-β-D-Glucoside (SSG) has elucidated its dual role in gouty arthritis. SSG not only mitigates the inflammatory response of macrophages by inhibiting the NF-κB and NLRP3 pathways, but it also downregulates the expression of FDX1 and DLAT in chondrocytes. Furthermore, it directly inhibits copper-induced cell death in chondrocytes within the inflammatory microenvironment [218]. This finding connects the two fundamental pathological processes of “inflammation and cell death,” suggesting that in autoimmune diseases, the inhibition of copper-induced cell death in specific cells may represent a novel strategy for tissue protection and disease progression delay.

Moreover, the mechanisms by which certain natural products exert their effects extend beyond the regulation of the classic FDX1-DLAT axis. Columbianadin (CBN) has been shown to stabilize the palmitoylase ZDHHC5 through its interaction with TRIM7, which enhances the membrane localization and copper excretion function of the P2X7 receptor, ultimately leading to the inhibition of copper-induced cell death in M2 macrophages within the synovial membrane [219]. This pathway underscores the crucial role of cell membrane receptors and the post-translational modifications of proteins in regulating copper homeostasis, thereby enhancing our understanding of the intricate cuproptosis regulatory network. Another case that merits thorough examination is 10-Hydroxy-2-decenoic acid (10-HDA). In cases of traumatic brain injury, copper homeostasis in the brain is not maintained through direct inhibition of the classic cuproptosis pathway. Instead, it is regulated by the upregulation of the copper excretion protein ATP7A, which subsequently inhibits the pyroptosis of neurons that is exacerbated by imbalances in copper homeostasis [220]. This suggests that the protective effect of natural products is not always realized through the direct “blocking” of cell death; rather, it may involve “stabilizing” the internal environment. This mechanism could be particularly significant in the context of complex injuries to the central nervous system.

**Figure 5:**
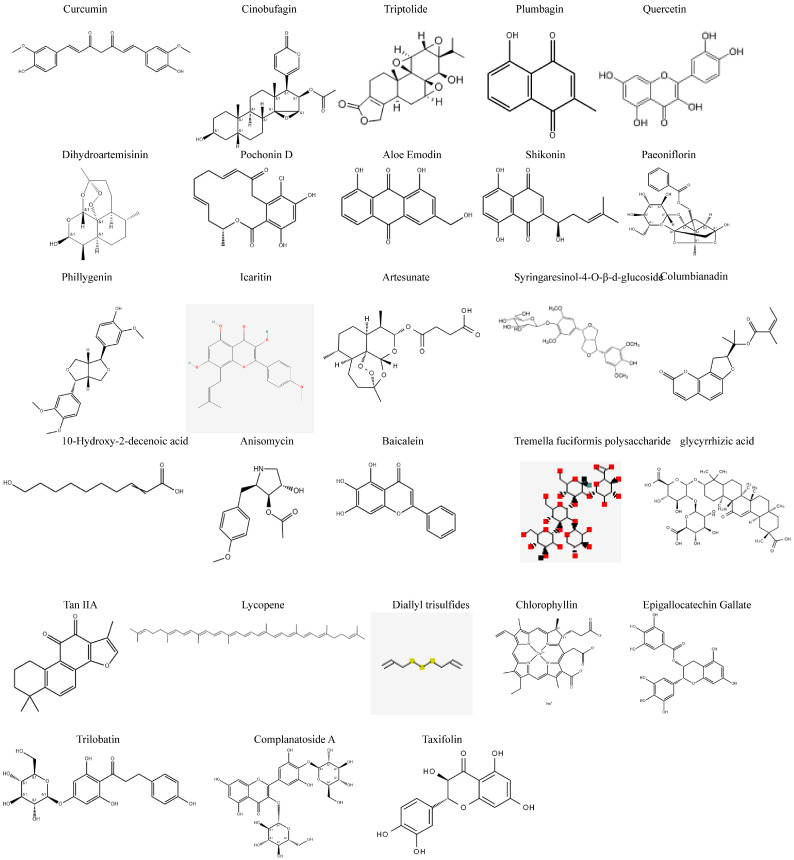
The chemical structure of natural products that regulate cuproptosis.

A review of existing research indicates that FDX1 serves as a central hub for the synergistic effects of various natural products. The methods of intervention are diverse, encompassing direct binding, the regulation of expression, and functional activation, which positions FDX1 as a highly attractive drug target. Additionally, tissue and disease specificity represent areas that require urgent and thorough investigation. For instance, why does quercetin inhibit copper-induced cell death in kidney injury while promoting it in liver cancer? This discrepancy may be attributed to differences in cell type-specific metabolic states, REDOX balance, and protein expression profiles. In conclusion, natural products, functioning as probes and lead compounds, have significantly enhanced our understanding of the physiological and pathological implications of copper-induced cell death (Figure 5, Table 4).

### 7.4. Copper-Based Nanoformulations

Copper-based nanomedicines, through innovative material design, have attained precise spatiotemporal control of copper-induced cell death, marking a significant advancement in this field. These materials encompass over 20 distinct nano-preparations, including nearly CaCu@CS-GOx, DCP-TPP, EsCu@TCM, DE-Cu_4_O_3_ NPs, and 5FCN, each exhibiting unique characteristics (Table 5). In the realm of tumor treatment, various nanoplatforms have exhibited distinct advantages. The CaCu@CS-GOx system employs a synergistic approach that combines copper-induced cell death and calcium overload, which is further enhanced by glucose deprivation, utilizing bimetallic Ca/Cu nanostructures [226]. DCP-TPP employs fatty acid camouflage to selectively target breast cancer cells that exhibit high CD36 expression, facilitating the precise release of disulfiram and copper ions under the control of near-infrared light [227]. EsCu@TCM achieves targeted copper delivery through a mitochondrial targeting design activated by near-infrared light [228]. DE-Cu_4_O_3_ nanoparticles demonstrate a marked inhibitory effect on non-small-cell lung cancer stem cells [229]. 5FCN persistently induces cell death in triple-negative breast cancer through a self-amplifying cycle of ferroptosis and copper-ptosis [230]. MC@BSA increases the sensitivity of tumor cells to copper-induced death by inhibiting the PKM2/HIF-1α/DLAT signaling axis [231]. Noteworthy are several ingeniously designed novel nanoplatforms: cystine-modified lignin–copper coordination nanocarriers release TKI inhibitors in response to both pH and glutathione, while simultaneously inducing copper-mediated cell death [232]. Two-dimensional copper-based piezoelectric metal–organic framework (MOF) sonosensitizers enhance the efficacy of sonodynamic therapy by harnessing the piezoelectric effect, while concurrently inducing copper-mediated cell death [233]. Ce6@Cu nanoparticles facilitate a sonodynamic-triggered combined treatment that targets both copper and iron death pathways in response to injury [234]. Cu(I)NP demonstrates stimulus response release characteristics and elicits an anti-tumor immune response injury [235]. siAtox1/ES@OMV facilitates the combined effects of copper homeostasis disruption and immune regulation via the outer membrane vesicle delivery system [236]. In the domains of anti-infection and immune regulation, copper-based nanoformulations also exhibit remarkable efficacy. Cu(DDC)_2_@BSA induces mitochondrial dysfunction in fungal infections by decreasing ATPase activity and mitochondrial membrane potential [237]. Au@MSN-Cu/PEG/DSF facilitates photothermal-triggered on-demand drug release for injury treatment [238]. Cu_2_O@SiO_2_-Ce6 integrates copper-induced cell death with photodynamic therapy [239]. Cu_2_O@Ce-MOFs/KL-11743 improves intracellular copper retention in injury through dual metabolic regulation [240]. TSF@ES-Cu NPs enables targeted therapy for pancreatic cancer by utilizing natural RGD peptides [241]. T-T@Cu exhibits the ability to target tumor mitochondria, while Cu@Fh innovatively integrates photoimmunotherapy with photodynamic therapy [242,243]. These designs not only improve the targeted delivery efficiency of copper but also facilitate multi-level and multi-pathway anti-tumor effects by coordinating with various death mechanisms and therapeutic modalities, including photothermal, sonodynamic, chemical, and immunotherapy.

### 7.5. Other Drugs

Additionally, various chemically synthesized drugs and metal complexes have been identified as exhibiting unique therapeutic potential in oncology, cardiovascular, and neurological diseases through the precise regulation of the cuproptosis pathway. Although these drugs operate through distinct mechanisms, collectively they underscore the promising prospects of cuproptosis as a therapeutic target.

In tumor treatment, the strategies involving chemical drugs exhibit notable diversity. Certain drugs function by directly inducing cuproptosis. Zinc pyrithione (ZnPT), an anti-tumor agent, was unexpectedly discovered to disrupt copper homeostasis in triple-negative breast cancer cells during screening. This disruption promotes the oligomerization of the key protein DLAT, ultimately leading to cuproptosis injury [244]. The case of “reusing old drugs” indicates that additional potential cuproptosis inducers may be concealed within approved drug libraries. In contrast, 4-Octyl Itaconate (4-OI), a derivative of itaconic acid, presents an innovative approach termed “metabolic sensitization.” Although it does not directly induce cuproptosis, 4-OI inhibits the key glycolytic enzyme GAPDH, compelling cancer cells to depend more on mitochondrial respiration. This shift enhances their susceptibility to subsequent cuproptosis induced by Elesclomol-Cu [245]. In the realm of tumor treatment, the strategies employed by chemical drugs exhibit remarkable diversity. Certain drugs directly induce cuproptosis. For instance, zinc pyrithione (ZnPT), an anti-tumor agent, was unexpectedly discovered to disrupt copper homeostasis in triple-negative breast cancer cells during screening, promoting the oligomerization of the critical protein DLAT and thereby inducing cuproptosis injury [246]. This presents a novel approach to addressing the issue of drug resistance stemming from tumor metabolic heterogeneity. Nonetheless, the intricate nature of the tumor microenvironment (TME) presents significant challenges to effective treatment. The targeted drug Lenvatinib, when administered for hepatocellular carcinoma, unexpectedly triggers the formation of neutrophil extracellular traps (NETs) via the IL33/PADI4 axis. These NETs have the capacity to inhibit copper-induced cell death in tumor cells, thereby contributing to drug resistance [247]. In contrast, the combination therapy of Docetaxel and Elesclomol-Cu has exhibited notable synergistic effects. This regimen significantly increases the sensitivity of prostate cancer to chemotherapy by upregulating DLAT and inhibiting mTOR-mediated autophagy, thereby arresting the cell cycle at the G2/M phase. It presents a robust strategy for overcoming chemotherapy resistance [248].

In the realm of protection against non-tumor diseases, the primary objective of chemical drugs has transitioned to the inhibition of cuproptosis to preserve normal tissues and organs. Within cardiovascular medicine, the hypoglycemic agent Dapagliflozin has exhibited cardiovascular protective effects that extend beyond its hypoglycemic properties. One mechanism underlying this effect involves the reduction of cuproptosis in myocardial cells following myocardial infarction, achieved through the inhibition of the HIF-1α/TGF-β signaling pathway [249]. Likewise, the antihypertensive agent Aprocitentan and the PCSK9 inhibitor Evolocumab effectively inhibited copper-induced cell death in cardiomyocytes within models of doxorubicin cardiotoxicity and myocardial ischemia–reperfusion injury. This inhibition occurred through the activation of SIRT7 by Aprocitentan and the disruption of the interaction between PCSK9 and LIAS by Evolocumab, respectively [250,251]. These findings not only elucidate novel pharmacological mechanisms of action for these drugs but also strongly indicate that copper-induced cell death is a critical factor in cardiovascular and cerebrovascular injuries. Regarding neuroprotection, magnesium hexacyanoferrate (MgHCF) and Edaravone dextrose (EDB) effectively chelate excess copper and iron ions while simultaneously downregulating CTR1 and FDX1. In models of sepsis-related encephalopathy and cerebral infarction, these agents reduced copper-induced neuronal death and improved cognitive function and nerve integrity. This presents new hope for the treatment of acute nerve injury, an area currently lacking effective therapeutic options [252,253].

Researchers have developed novel inhibitors and inducers that specifically target the cuproptosis pathway. Merestinib (MTB) has emerged as the first small molecule compound that can directly inhibit cuproptosis. This compound alleviates acute liver injury in the model by binding to and activating the antioxidant core transcription factor NRF2, while also promoting copper excretion. Its discovery offers hope for treating diseases associated with excessive activation of cuproptosis [254]. Conversely, the engineered Cu(II)-Dipyridophenazine complex, along with other metal-based therapeutics, adeptly combines several functions, including the consumption of glutathione (GSH), the generation of reactive oxygen species (ROS), and the release of copper ions to induce copper-mediated cell death. This approach exemplifies a synergistic strategy that merges chemokinetic therapy with copper-induced cytotoxicity, marking a progressive direction in the design of drugs for tumor treatment [255].

The mechanisms and therapeutic potential of natural compounds and their nanocarriers in regulating cuproptosis continue to require extensive investigation. Taking quercetin as an example, its copper-complexed form (CuQ) not only exhibits superior antioxidant activity compared to free quercetin but can also switch to a pro-oxidant state in the tumor microenvironment [256], inducing mitochondrial oxidative damage and DNA strand breaks. This suggests its ability to disrupt copper homeostasis and energy metabolism, thereby activating cell death pathways including cuproptosis. Further studies indicate that the introduction of nanocarriers such as liposomes significantly enhances the stability and targeted delivery efficiency of such natural compounds. Nano-curcumin liposomes, for instance, effectively downregulate the expression of FDX1—a key regulator of cuproptosis—in gastric adenocarcinoma cells while suppressing the antioxidant protein GPX4 [257]. This dual action cooperatively disrupts mitochondrial function, intensifies oxidative stress, and promotes cuproptosis-like programmed cell death in tumor cells. Moreover, such nano-formulations can modulate molecules involved in cell migration and metabolic reprogramming, such as SERPINE1 and SLC27A5, thereby inhibiting tumor progression through multiple pathways. These findings not only reveal the synergistic mechanism of the natural compound–copper–nanocarrier ternary system in precisely regulating cellular cuproptosis responses, but also underscore its profound implications for therapeutic intervention in pathologies such as cancer, inflammatory diseases, and tissue regeneration via remodeling of metal homeostasis and metabolic networks.

## 8. Summary

We systematically reviewed the latest research progress on cuproptosis as a novel form of programmed cell death, including its molecular mechanisms, signaling pathways, role in diseases, and targeted therapeutic strategies. Research has found that the core mechanism of cuproptosis lies in copper ion overload within mitochondria. Through FDX1-dependent reduction reactions, it leads to functional oligomerization and aggregation of acylated TCA cycle enzymes, thereby triggering protein toxic stress. This process is precisely regulated by a complex signaling network including HIF-1α, Nrf2, PI3K/AKT, cGAS-STING, etc., and is closely related to the functions of various subcellular organelles such as mitochondria, lysosomes, and endoplasmic reticulum. At the disease level, cuproptosis has been proven to be widely involved in a variety of major pathological processes such as tumors, neurological diseases, cardiovascular diseases, metabolic diseases, and immune diseases. The related genes and proteins have become highly potential diagnostic and prognostic biomarkers. In the field of therapy, in addition to traditional copper chelating agents and copper ion carriers, ingeniously designed copper-based nanomedicines have achieved precise spatiotemporal regulation of copper mortality. Meanwhile, the discovery of a large number of natural products has revealed broad prospects for bidirectional intervention of copper mortality through direct targeting of FDX1 and regulation of copper homeostasis. Despite considerable advancements in research, this field continues to encounter numerous challenges. First, regarding mechanistic studies, our current understanding of cuproptosis remains predominantly focused on the FDX1-DLAT axis. It remains uncertain whether additional parallel or cell type-specific pathways for cuproptosis induction exist. Furthermore, the interplay between cuproptosis and other cell death modalities, including ferroptosis, apoptosis, pyroptosis, and even pan-apoptosis, requires urgent investigation. Cuproptosis differs fundamentally from other programmed cell death modalities—such as ferroptosis, apoptosis, pyroptosis, and necroptosis—in its core mechanism, yet it shares certain pathophysiological contexts with them. Its uniqueness lies in the direct triggering by excessive accumulation of Cu^+^ within mitochondria and the specific targeting of lipoylated metabolic enzymes in the tricarboxylic acid cycle, leading to the loss of enzyme function, protein aggregation, and mitochondrial metabolic collapse. Unlike ferroptosis, which depends on lipid peroxidation and GPX4 inactivation; apoptosis, driven by caspase cascade activation; pyroptosis, mediated by gasdermin-dependent pore formation; or necroptosis, which relies on the RIPK1/RIPK3/MLKL signaling axis, cuproptosis operates independently of these pathways. Nevertheless, these forms of cell death are interconnected, as they can all be induced by disturbances in metal homeostasis, oxidative stress, and organelle dysfunction and may intersect or synergize under certain pathological conditions. Secondly, the absence of highly sensitive and specific dynamic monitoring tools for in vivo cuproptosis significantly hampers the real-time evaluation of its role in both physiological and pathological processes. Moreover, existing therapeutic strategies involving cuproptosis inducers, such as Elesclomol, demonstrate inadequate tumor targeting and may lead to systemic toxicity. The primary challenge in future drug development lies in achieving precise regulation of cuproptosis across various disease contexts.

## Figures and Tables

**Table 1:** Signaling pathways related to cuproptosis.

Signal Pathway	Disease	Mechanism	Key Target	References	Type
HIF-1α	MI	Inhibit HIF-1α/TGF-β signaling, reduce copper accumulation and ROS, and alleviate myocardial fibrosis mediated by cuproptosis.	HIF-1α, TGF-β	[73]	original research
	CC	Overexpression of VIPR1 inhibits HIF-1α signaling, enhances cuproptosis sensitivity, and suppresses tumor cell proliferation.	HIF-1α, VIPR1, FDX1, DLST	[74]	original research
ATOX1	DLBCL	ATOX1 promotes cell proliferation through the MAPK pathway. Its knockdown can enhance the sensitivity to cuproptosis, leading to cell cycle arrest.	ATOX1, ERK1/2	[81]	original research
COMMD1/ATP7A	HIE	Hypoxia disrupts the COMMD1/ATP7A axis, leading to abnormal accumulation of copper ions, inducing cuproptosis and causing neuronal damage.	COMMD1, ATP7A	[85]	original research
C5a/C5aR	BC	The activation of the C5a/C5aR pathway upregulates integrin ATP7B through the Wnt/β-catenin signaling, promoting copper excretion and leading to cuproptosis resistance.	C5a, C5aR, β-catenin, ATP7B	[88]	original research
SPI1/CDKN2A/p53	LUAD	The downregulation of the transcription factor SPI1 relieves the inhibition of CDKN2A and activates the p53 signal, thereby enhancing the cell’s sensitivity to cuproptosis	SPI1, CDKN2A, p53	[91]	original research
ERK	CI	polystyrene nanoplastics induce oxidative stress to activate the ERK-MAPK pathway, leading to copper accumulation and cuproptosis in neurons.	ERK, MAPK	[92]	original research
	HCC	SEC14L3 positively regulates cuproptosis through the ERK/YY1/FDX1 signaling axis, thereby inhibiting tumor tissue growth	ERK, YY1, FDX1, SEC14L3	[93]	original research
cGAS/STING	Melanoma, BC	cuproptosis leads to the release of mtDNA, activates the cGAS-STING pathway, induces immunogenic cell death, and enhances anti-tumor immunity.	mtDNA, cGAS, STING	[83,84]	original research
PI3K	CRC, SIONFH	After PI3K activates AKT, it inhibits FDX1, promotes metabolic reprogramming, inhibits cuproptosis, and reduces drug resistance.	PI3K, AKT, mTOR, FDX1	[76,78]	original research
	OSCC	Overexpression of PER2 reduces the interaction between HSP70 and AKT, inhibits the AKT pathway and promoting cuproptosis.	PER2, HSP70, AKT, DLAT, CTR1	[77]	original research
	CI/RI	Inhibit GSK-3β, reduce ATF3 expression, thereby downregulating the copper transporter CTR1 and reducing copper accumulation.	PI3K, GSK-3β, ATF3, CTR1	[79]	original research
Wnt/β-catenin	BC	The β-catenin/TCF4 complex transcriptionally upregulates ATP7B, promotes copper excretion, forms a negative feedback loop, and reduces drug resistance.	β-catenin, TCF4, ATP7B	[96]	original research
Nrf2	TBI	circSpna2 binds to Keap1, preventing the degradation of Nrf2. Nrf2 entering the nucleus upregulates the expression of ATP7B, promotes copper excretion, and alleviates cuproptosis and neuronal damage.	circSpna2, Keap1, Nrf2, Atp7b	[72]	original research

**Annotation:** MI: myocardial infarction; CC: colon cancer; DLBCL: diffuse large B-cell lymphoma; HIE: hypoxic–ischemic encephalopathy; BC: breast cancer; LUAD: lung adenocarcinoma; CI: cognition impairment; HCC: hepatocellular carcinoma; CRC: colorectal cancer; SIONFH: osteonecrosis of the femoral head; OSCC: oral squamous cell carcinoma; CI/RI: cerebral ischemia/reperfusion injury; TBI: traumatic brain injury; VIPR1: vasoactive intestinal peptide receptor 1.

**Table 2:** Biomarkers and mechanisms of cuproptosis in tumors.

Name	Biomarker	Mechanism	References	Type
Glioma	FDX1, LIPT2, NNAT	High expression of FDX1 mediates cuproptosis by regulating protein thioacylation	[98]	original research
Lung cancer	DBH, UBE2D3, SOD1, UBE2D1, LOXL2	Interfering with the TCA cycle leads to the accumulation of Cu ions and increases the level of oxidative stress	[108]	database research
Lymphoma	ATOX1, SLC11A2, MT1H, MT1X, MT2A	ATOX1 inhibits proliferation and promotes cell cycle arrest by controlling the MAPK pathway through copper transport	[94]	original research
Liver cancer	CDKN2A, DLAT, GLS, LIPT1, MTF1, G6PD, PRR11, KIF20A, EZH2, CDCA8	Interfering with the TCA cycle leads to energy metabolism disorders, increases oxidative stress and inflammation levels, and promotes the infiltration and growth of tumor tissues	[109]	original research
Breast cancer	CDKN2A, MTF1, PDHA1, DLD, LIPT1, FDX1, CTR1	The copper influx mediated by CTR1 and the Cu^2+^ reduction catalyzed by FDX1 jointly lead to the aggregation of toxic Cu^+^ with LIPT1/LiAS-dependent acylated proteins, thereby triggering the collapse of the tricarboxylic acid cycle and mitochondrial stress	[110]	database research
Bladder cancer	PDGFRB, COMP, GREM1, FRRS1, SDHD, RARRES2, CRTAC1, HMGCS2	Regulate the sensitivity of cells to cuproptosis through indirect pathways such as influencing cell proliferation, mitochondrial function or stress response	[111]	database research
Head and Neck Squamous Cell Carcinoma	ISCA2, CAT, MTFR1L, OXAL1L	The sensitivity of cells to cuproptosis can be regulated by regulating mitochondrial iron–sulfur cluster biosynthesis, eliminating hydrogen peroxide to alleviate lipid peroxidation, or influencing mitochondrial morphology and function	[112]	database research
Renal cancer	CDKN2A, FDX1, LIAS, DLAT	Reducing copper ions and catalytic acylation modification provide toxic targets for copper, disrupting the tricarboxylic acid cycle	[113]	database research
Endometrial cancer	SCG2, CKMT1B, MACC1, ADAMTS16, NTS, DCAF12L1, LMO3, THRB, SIX1, SHISA9, CDKN2B, ARFGAP3,H3C1	Regulating indirect ways such as cell signal transduction, metabolic reprogramming, epigenetic modification or cell cycle to affect the sensitivity of cells to cuproptosis	[114]	database research
Prostate cancer	FDX1, PDHA1, MTF1, CDKN2A	Disrupting the TCA cycle and promoting protein acylation directly drives cell death	[115]	database research
Gastric cancer	CIAO1, ACLY, FGD6, SERPINE1, SPATA13, RANGAP1, ADGRE5	Regulate cell metabolism, cell adhesion and migration or signal transduction pathways, and affect the sensitivity of cells to cuproptosis	[116]	original research
Cervical cancer	COX7B, PIH1D2, NDUFA1, NDUFA2, SLC25A5, SLC6A3	Interfere with the transmitochondrial matrix transport of ATP and ADP within the mitochondrial matrix, causing energy metabolism disorders and the accumulation of copper ions	[117]	database research
Colon cancer	CDKN2A, SDHB, CCS, ULK1, CMC1, GLS, NOX1, HOXC6, TNNT1, PLA2G12B; COX17, DLAT	The homeostasis of copper ions is unbalanced, with an increase in Cu^+^ generation, which disrupts the TCA cycle, raises the level of oxidative stress, and affects glutamine metabolism and autophagy function	[118]	database research
Esophageal cancer	CDKN2A, PDHA1, LIAS, DLAT, FDX1, BTLA, CT47A1, PRRX1	Reduce copper ions and drive protein acylation, disrupt the TCA cycle, and affect the cell cycle	[119]	database research
Ovarian cancer	TIMM8B, COX8A, SSR4, HIGD2A, WASF2, PRDX5, CLDN4	Interfering with the mitochondrial respiratory chain affects energy metabolism and enhances antioxidant responses	[120]	original research
Oral cancer	PLAU, IL1A, SPP1, CCL11, TERT, COL1A2, GLS, MTF1	It catalyzes the hydrolysis of glutamine to provide metabolic substrates for cuproptosis-dependent mitochondrial respiration and regulates copper homeostasis to resist excessive accumulation of copper ions	[121]	database research
Skin cancer	LIPT1, PDHA1, CTR1, ORAI2, ACADSB, SLC47A1; SNAI2, RAP1GAP	It affects glutamine metabolism, leading to mitochondrial respiratory disorders, and increases Cu+ production, resulting in increased copper toxicity	[122]	database research
Laryngeal cancer	TMEM2, DACT1, STMN2, GPR173	Regulate the Wnt and TGF-β signaling pathways, influencing cytoskeleton dynamics or extracellular matrix remodeling	[123]	database research
Bone cancer	ATP7A, FDX1, PDHA1, PDHB, MTF1, CDKN2A, DLST	Drive the toxicity of copper-dependent acylated proteins, disrupt the TCA cycle, promote the extracellular transport of copper, and cause copper accumulation	[124]	database research
Leukemia	MTF1, LIPT1	MTF1 regulates metallothionein to mediate copper excretion in cells to resist cuproptosis, while LIPT1 directly generates copper toxicity targets by participating in protein lipoacylation modification to drive cuproptosis	[125]	database research
Thyroid cancer	CTR1, LIAS, DLD, MTF1, CDKN2A, GCSH	The copper influx mediated by CTR1 leads to copper accumulation. LIAS and GCSH synergistically catalyze the thioocylation of the protein, while MTF1 resists this process by activating copper detoxification genes	[126]	database research
Pancreatic cancer	AKR1B10, KLHL29, PROM2, LIPT1, LIAS, PDP1, GCSH	Promote the direct generation of copper-toxic targets through lipoylation modification of proteins, as well as by regulating the activity of the pyruvate dehydrogenase complex	[127]	original research
Cholangiocarcinoma	ADAM9, ADAM17, ALB, AQP1, CDK1, MT2A, PAM, SOD3, STEAP3, TMPRSS6	MT2A resists copper toxicity by chelating copper ions, and STEAP3 promotes the formation of toxic Cu^+^ by mediating copper reduction	[128]	database research

**Table 3:** Biomarkers and mechanisms of cuproptosis in non-tumor diseases.

System	Disease	Biomarkers	Mechanism	References	Type
Nervous	Stroke	NLRP3, NFE2L2, ATP7A, LIPT1, GLS, MTF1	Copper transport disruption leads to mitochondrial metabolic dysfunction, abnormal lipoylated protein accumulation activates the inflammasome and exacerbates oxidative stress.	[129,130]	database research
	Alzheimer’s Disease	IFI30, PLA1A, ALOX5AP, A4GALT, FDX1, DLD, DLAT, PDHA1, PDHB, GLS, LIPT1, CDKN2A, CTR1, ATP7B	Copper accumulation drives FDX1-dependent lipoylation toxicity, disrupts TCA cycle and energy metabolism, promotes neuronal mitochondrial dysfunction and inflammatory responses.	[131]	database research
	Amyotrophic Lateral Sclerosis	BAP1, SMG1, BCLAF1, DHX15, EIF4G2	Dysregulated RNA metabolism and copper homeostasis synergistically induce mitochondrial stress, promoting motor neuron death.	[135]	original research
	Parkinson’s Disease	SLC18A2, SLC6A3, SV2C	Dysregulated synaptic vesicle copper transport in dopaminergic neurons impairs neurotransmitter release and induces oxidative damage.	[136]	database research
	Major Depressive Disorder	OSBPL8, VBP1, MTM1, ELK3, SLC39A6	Zinc-copper ion imbalance affects neurotransmitter signaling and mitochondrial function, exacerbating neuronal metabolic stress.	[137]	database research
	Epilepsy	LIPT1, GLS, PDHA1, CDKN2A, DLD, FDX1, DLAT, PDHB	Energy metabolism dysfunction and copper-dependent lipoylated protein aggregation jointly lead to neuronal excitation-metabolism coupling dysregulation.	[138]	original research
Musculoskeletal	Sarcopenia	PDHA1, PDHB, DLAT, DLST, DLD, FDX1, LIAS, NDUFC1	Abnormal lipoylation of TCA cycle key enzymes causes mitochondrial energy failure, promoting muscle cell atrophy and apoptosis.	[152,153]	database research
	Intervertebral Disc Degeneration	LIPT1, DLAT, PDHB, GCSH, DLST, ATP7A, ATP7B, MTF1	Dysregulated copper efflux in disc cells leads to metal accumulation, inducing mitochondrial lipoylated protein toxicity and extracellular matrix degradation.	[154]	original research
	Osteoarthritis	DBT, LIPT1, GLS, PDHB, FDX1, DLAT, PDHA1	Copper toxicity in chondrocytes causes TCA cycle arrest and antioxidant defense collapse, accelerating cartilage destruction.	[155]	database research
	Rheumatoid Arthritis	FAM96A, CGRRF1	Cross-talk between iron–sulfur cluster assembly and cuproptosis pathways promotes synovial cell inflammation and apoptosis.	[156]	database research
	Spinal Cord Injury	DLD, ATP7A, CP, LOXL2, PDE3B	Copper accumulation inactivates lipoylated dehydrogenases, impairs energy metabolism and exacerbates secondary neural tissue damage.	[157]	database research
	Osteoporosis	EVA1B, RTCB, HEXB, SLC25A11, TMEM126B	Mitochondrial metabolic dysfunction and copper transport abnormalities in osteoblasts collectively reduce bone formation capacity.	[158]	database research
Digestive	Crohn’s Disease	ABCB6, BACE1, FDX1, GLS, LIAS, MT1M, PDHA1, ETC. (25 CUDEGS)	Widespread dysregulation of cuproptosis-related genes in intestinal epithelial cells leads to mucosal barrier metabolic collapse and immune activation.	[148]	database research
	Inflammatory Bowel Disease	PDHA1, DBT, DLAT, LIAS	Copper-induced disruption of lipoylation in energy metabolism key enzymes exacerbates intestinal inflammation and epithelial injury.	[149]	database research
	Ulcerative Colitis	ATOX1, SUMF1, MT1G, ATP7B, FDX1, LIAS	Dysregulation of copper chaperones and the lipoylation system synergistically leads to colonic epithelial mitochondrial failure and oxidative damage.	[150]	database research
Cardiovascular	Myocardial Infarction	LIAS, LIPT1, DLAT, PDHB, MTF1, GLS	Activation of cuproptosis pathway in cardiomyocytes interrupts TCA cycle, causing energy supply failure and promoting cardiomyocyte necrosis.	[140]	database research
	Dilated Cardiomyopathy	SEPTIN1, CLEC11A, ISG15, P3H3, SDSL, INKA1	Cytoskeletal-associated proteins and immune signals co-regulate mitochondrial copper sensitivity, promoting myocardial remodeling.	[142]	database research
	Primary Cardiomyopathy	MAP2K1, FDX1, CTR1	Increased copper uptake and FDX1 overactivation synergistically induce mitochondrial lipoylation toxicity, leading to myocardial metabolic disorder.	[143]	original research
	Atherosclerosis	FDX1, CTR1, GLS	Copper-dependent metabolic reprogramming in vascular endothelial cells promotes oxidative stress and inflammatory plaque formation.	[144]	original research
	Myocardial Ischemia–Reperfusion Injury	DLAT, PDHB, PDHA1	Copper-mediated inactivation of TCA cycle enzymes during reperfusion exacerbates myocardial energy crisis and cell death.	[145]	database research
Immune	Sepsis	LIAS, PDHB, GLS, CD274, CP, VEGFA, ETC.	Immune metabolic reprogramming and cuproptosis cross-activation promote macrophage mitochondrial dysfunction and amplify systemic inflammation.	[159]	database research
	Sjögren’s Syndrome	EED, CBL, NFU1	Epigenetic regulation and abnormal iron–sulfur cluster synthesis collectively influence salivary gland cell susceptibility to cuproptosis.	[160]	database research
	Systemic Lupus Erythematosus	LIAS, CDKN2A	Abnormal lipoylation metabolism and cell cycle regulation synergistically promote mitochondrial dysfunction in autoimmune T cells.	[161]	database research
	Tuberculosis	NFE2L2, NLRP3, ATP7B, CTR1, MTF1, DLD, LIAS, LIPT1, DLAT, GLS, DBT	Copper as an antibacterial weapon triggers host cell lipoylation metabolic collapse and inflammatory storm in macrophages.	[162]	database research
	Behçet’s Disease	ANKRD9, COX11, MT1G, MT2A, MT4, TYR	Compensatory upregulation of metallothionein system is insufficient to counteract copper-induced mitochondrial toxicity, leading to vascular endothelial injury.	[163]	database research
	Allergic Rhinitis	MRPS30, CLPX, MRPL13, MRPL53	Mitochondrial ribosomal protein dysfunction and copper stress synergistically exacerbate respiratory mucosal immune-metabolic imbalance.	[164]	database research
Respiratory	Idiopathic Pulmonary Fibrosis	AKAP9, ANK3, C6ORF106, LYRM7, MBNL1	Mitochondrial copper homeostasis dysregulation and abnormal RNA splicing jointly promote fibroblast aberrant activation and pulmonary fibrosis.	[170]	original research
	Asthma	RIM25, DYSF, NCF4, ABTB1, CXCR1	Copper-related oxidative burst and airway hyperresponsiveness form a positive feedback loop in immune cells.	[171]	original research
	Pneumonia	ATP7B, DBT, DLAT, DLD, FDX1, GCSH, LIAS, LIPT1, CTR1	Pathogen infection and cuproptosis pathway co-activation lead to alveolar epithelial cell metabolic collapse and barrier function loss.	[172]	database research
	Pulmonary Arterial Hypertension	AHR, FAS, FGF2	Vascular remodeling signals and cuproptosis synergistically promote proliferation-apoptosis imbalance in pulmonary artery smooth muscle cells.	[173]	database research
Endocrine & Metabolic	Non-alcoholic Fatty Liver Disease	NFE2L2, DLD, POLD1, PDHB	Hepatic lipid metabolism disorder and copper-mediated TCA cycle enzyme inactivation jointly drive steatosis progression to inflammation and fibrosis.	[167]	database research
	Alcoholic Hepatitis	ALDOA, COL3A1, LUM, THBS2, TIMP1	Alcohol and copper synergistically disrupt hepatocyte metabolic balance, accelerating extracellular matrix deposition and liver injury.	[168]	original research
	Diabetes Mellitus	CTR1, ATP7A, FDX1	Increased copper uptake in pancreatic β-cells induces FDX1-dependent lipoylation toxicity, leading to insulin secretion dysfunction.	[169]	original research
Other Diseases	Endometriosis	PDHA1	PDHA1-mediated glycolytic abnormalities in ectopic endometrial cells enhance cuproptosis susceptibility, promoting lesion survival.	[174]	database research
	Polycystic Ovary Syndrome	COL5A1, IL18BP, SLC12A5, MDK, RXRG	Metabolic–inflammatory cross-talk and copper homeostasis imbalance in theca cells collectively promote ovarian dysfunction.	[175]	database research
	Psoriasis	MTF1, ATP7B, CTR1	Compensatory enhancement of copper efflux function in keratinocytes is insufficient to reverse cuproptosis-associated epidermal hyperproliferation and inflammation.	[176]	database research
	Renal Ischemia–Reperfusion Injury	LIPA, LIPT1, SDHB, NDUFB6, MOAP1, PPP2CA, SYL2, ZZZ3, SFRS2	Cross-activation of cuproptosis and ferroptosis pathways in renal tubular epithelial cells synergistically amplifies acute kidney injury.	[177]	original research
	Diabetic Nephropathy	FSTL1, CX3CR1, AGR2	Metabolic memory and copper toxicity jointly mediate podocyte mitochondrial dysfunction, accelerating glomerulosclerosis.	[178]	original research
	Periodontitis	MTF1, GLS, DLST	Copper stress and glutamine metabolic remodeling in gingival epithelial cells collectively disrupt the oral mucosal barrier and exacerbate local inflammation.	[179]	original research
	Chronic Renal Fibrosis	BCL2A1B, CLEC4N, COL1A1	Immune cell infiltration and fibroblast activation synergistically promote renal fibrosis in a copper-enriched microenvironment.	[181]	original research

**Table 4:** Research on component–target–mechanism analysis of natural product regulation of cuproptosis.

Name	Source	Model	Target/Pathway	Mechanism	References
Curcumin	*Curcuma longa*	Liver and cervical cancer cells	FDX1, GSH; Notch1/RBP-J/NRF2 pathway	As a copper ion carrier to increase copper accumulation within cells; or inhibit Notch1/RBP-J, downregulate NRF2, upregulate FDX1, and induce cuproptosis.	[195,221]
Baicalein	*Scutellaria baicalensis*	SiHa, HeLa cells	FDX1, SDHB; PI3K/Akt pathway	By inhibiting the Akt signaling pathway to upregulate the expression of FDX1 and SDHB, cuproptosis is induced and cisplatin sensitivity is enhanced.	[222]
Triptolide	*Tripterygium wilfordii*	HeLa, SiHa cells	XIAP/COMMD1/ATP7A/B pathway; FDX1, LIAS, DLAT	Downregulating ATP7A/B and upregulating CTR1 increase intracellular copper accumulation and induce cuproptosis.	[200]
Quercetin	A variety of plants	AKI model mice, HK-2 cells, HCC cells	FDX1, ATP7B, GLS; SLC7A11/GPX4	Downregulating ATP7B/GLS in nephropathy inhibits copper mortality. In liver cancer, FDX1 is combined to enhance mitochondrial function and synergistically induce cuproptosis with ES-Cu.	[216]
Tremella fuciformis polysaccharide	*Tremella fuciformis*	K7M2 cells, RAW264.7 macrophages; Tumor-bearing mice	Cu^2+^, TNF-α, IFN-γ, IL-6, IL-1β	It coordinates with copper to form an intelligent gel, releasing copper ions in the tumor microenvironment, inducing cuproptosis and promoting the polarization of M1-type macrophages.	[223]
Glycyrrhizic acid	*Glycyrrhiza glabra*	Tumor microenvironment model	Cu^2+^, GSH, ROS	It self-assembled with norcantharidin into a hydrogel, generating reactive oxygen species through Cu^2+^ -mediated photodynamics, consuming GSH, and synergistically inducing cuproptosis and apoptosis.	[224]
Cinobufagin	*Bufo gargarizans*	HepG2, HUH7 cells	CTR1/CTR2, LIAS, ATP7A/B	Upregulate copper import protein (CTR1/2) and LIAS, downregulate excrete protein (ATP7A/B), increase intracellular copper accumulation and ROS, and induce cuproptosis.	[199]
Eupalinolide B	*Eupatorium lindleyanum*	Pancreatic cancer cells; Transplanted tumors in nude mice	Cu^2+^, ROS	Disrupt the copper homeostasis, increase ROS, and work in synergy with the copper ion carrier elesclomol to induce cuproptosis.	[202]
Qiju Dihuang Pill	*Lycii Fructus*, *Chrysanthemi Flos*, *Rehmanniae Radix Praeparata*, *Corni Fructus*, *Moutan Cortex*, *Dioscoreae Rhizoma*, *Poria*, *Alismatis Rhizoma*	HLEC cells	CTR1, FDX1, m6A modification	By reducing the m6A modification stability of CTR1 mRNA, downregulating its expression, and decreasing copper intake, cuproptosis is inhibited.	[213]
Paeoniflorin	*Paeonia lactiflora*	AMI model rat	FDX1, DLAT, Cu^2+^	Reduce the expression of FDX1, DLAT and serum copper levels, increase pyruvate, inhibit cuproptosis and improve cardiac remodeling.	[209]
Icaritin	*Epimedium*	Cu-es-induced HT22 cells	FDX1, DLAT, ATP7B, CTR1	Downregulate FDX1 to inhibit DLAT oligomerization, adjust ATP7B, and restore copper homeostasis with CTR1.	[214]
Tanshinone IIA	*Salvia miltiorrhiza*	Mouse xenograft tumor	FDX1,METTL3/METTL14-mediated m6A modification	METTL3/METTL14 increases the m6A modification of FDX1 and induces the aggregation of acylated DLAT	[225]
Lycopene	*Solanum lycopersicum*	Liver injury model chicken, ATR induces hepatotoxic cells	ATOX1	Directly bind to ATOX1, inhibit ATR-induced cuproptosis in hepatocytes, and alleviate mitochondrial damage.	[80]
Columbianadin	*Angelica pubescens Maxim. f. biserrata Shan et Yuan*	Traumatic synovitis model mice	TRIM7/P2X7/ZDHHC5 pathway	Targeting TRIM7, it protects ZDHHC5-mediated P2X7 palmitoylation and promotes copper exfoliation.	[219]
Plumbagin	*Plumbago zeylanica*	HCC model cells	DNMT1/miR-302a-3p/ATP7B pathway	Reducing DNMT1 and upregulating miR-302a-3p, thereby downregulating ATP7B, leading to intracellular copper accumulation and inducing cuproptosis.	[201]
Artesunate	*Artemisia caruifolia Buch.-Ham. ex Roxb.*	Parkinson’s disease model mice	MT2A, FDX1, CTR1	Upregulate the expression of MT2A, reduce the intracellular Cu^2+^ level, and downregulate FDX1.	[213]
Dihydroartemisinin		CRC model cells	LOXL2, FDX1	Inhibit LOXL2, block its mediated glycerophospholipid metabolic reprogramming, and relieve the inhibition of cuproptosis.	[204]
Phillygenin	*Forsythia suspensa*	MI/RI model mice; H9c2 cells	FDX1, LIAS, CTR1	Downregulate FDX1 and LIAS, and promote the lysosomal degradation of the copper import protein CTR1, reducing copper accumulation	[210]
Syringaresinol-4-O-β-d-glucoside	*Eleutherococcus senticosus (Rupr. & Maxim.) Maxim.*	Gouty arthritis model mice	FDX1, DLAT, NF-κB/NLRP3 pathway	Downregulate FDX1 and DLAT to inhibit cuproptosis induced by MSU	[218]
10-hydroxy-2-decenoic acid	Royal jelly	TBI model mice	ATP7A, Cu^2+^	Regulate the expression of the copper transporter ATP7A to maintain copper homeostasis in the brain.	[220]
Diallyl trisulfides	*Allium sativum*	LX2 cells	RAB18, CPT1A, DLD	Induce RAB18 phase separation and promote MAM formation, selectively activating cuproptosis in hepatic stellate cells.	[217]
Chlorophyllin	*Chlorophyll*	PC cells	FDX1, DLAT, GSH, ROS	Depleting GSH, increasing ROS, promoting FDX1-mediated DLAT oligomerization, and inducing cuproptosis.	[203]
Epigallocatechin Gallate	*Camellia sinensis*	HCC cells	MTF1/ATP7B pathway	Inhibit the transcription factor MTF1, downregulate the copper efflux protein ATP7B, and promote intracellular copper accumulation.	[196]
Pochonin D	*Ilyonectria* sp.	TNBC cells	PRDX1, PoD	Directly bind to and inhibit the enzymatic activity of PRDX1.	[206]
Aloe Emodin	*Aloe vera*	LLC tumor-bearing mice	Cu^2+^, PDT	Cu^2+^ is loaded to form nanoparticles, which are released at the tumor site and directly induce cuproptosis.	[208]
Shikonin	*Lithospermum erythrorhizon*	Ovarian cancer cells	Cu^2+^, GSH, ROS	Intracellular reductive release of Shikonin and Cu^2+^ synergistically triggers ROS-dependent necrosis and copper-induced death.	[207]
Trilobatin	*Lithocarpus polystachyus Rehd*	H9c2 cells	FDX1	Directly binding to FDX1, it inhibits DoX-induced cuproptosis, alleviates mitochondrial oxidative stress and myocardial injury.	[211]
Complanatoside A	*Astragalus membranaceus*	Subcutaneous transplanted tumor model mice	ATOX1	Downregulate ATOX1, promote intracellular copper ion accumulation, inhibit mitochondrial activity, and induce cuproptosis.	[197]
Taxifolin	Onion	In vivo transplanted tumor model mice	CTR1	Upregulation of CTR1 increases intracellular copper intake and induces cuproptosis.	[198]

**Table 5:** The mechanism of nano-platform preparations in interfering with cuproptosis.

Name	Disease	Mechanism	References
CaCu@CS-GOx	Liver cancer	The acidic microenvironment triggers the release of Ca^2+^ and Cu. Through the synergy of glucose starvation, calcium overload and cuproptosis, it inhibits tumor stemness and reshapes the immunosuppressive microenvironment. Combined with aCTLA-4 therapy, it enhances anti-tumor immunity.	[226]
DCP-TPP	Breast cancer	Near-infrared light triggers the release of DSF and Cu^3^, generating CuET which disrupts the copper homeostasis, inducing DLAT aggregation, disrupting the TCA cycle and fatogenesis, leading to uncoupling of mitochondrial oxidative phosphorylation, and activating anti-tumor immunity.	[227]
EsCu@TCM	Ovarian cancer	Mitochondrial-targeted delivery of Elesclomol-Cu(II) promotes mitochondrial copper accumulation under near-infrared irradiation, disrupts membrane potential, depletes ATP, downregulates FDX1 and induces DLAT oligomerization, while inhibiting ATP7A to enhance intracellular copper retention.	[228]
DE-Cu_4_O_3_ NPs	Lung cancer	Induce mitochondrial dysfunction, resulting in loss of membrane potential and reduction in mtDNA copy number. By inhibiting the AKT pathway, it downregulates TERT/TRF1, triggering telomere collapse, and simultaneously inhibiting tumor stem cell markers.	[229]
5FCN	Triple-negative breast cancer	Release Cu^2+^ and consume GSH, accumulate Cu^+^, leading to the aggregation of lipidated proteins, and synergistically amplifying cuproptosis and iron death.	[230]
Cu(DDC)_2_@BSA	Fungal infection	Lowering the membrane potential and ATPase activity leads to copper ion overload, upregulation of HSP70 and downregulation of lipoic acid synthase, inducing a copper-like death-like antifungal effect.	[237]
Au@MSN-Cu/PEG/DSF	Breast, liver cancer	Thermal-hydrothermal triggering release of DSF and Cu^2+^, in situ formation of CuET, induces apoptosis and cuproptosis, and cooperates with photothermal therapy to kill tumor cells.	[238]
Cu_2_O@SiO_2_-Ce6	Breast cancer, melanoma	The acidic microenvironment releases copper ions, inducing mitochondrial cuproptosis; at the same time, combined with photodynamic therapy, it generates reactive oxygen species, achieving dual killing effects.	[239]
MC@BSA		By inhibiting copper efflux and promoting the production of ROS to disrupt copper homeostasis, it leads to the aggregation of lipidated proteins and ATP depletion; inhibiting the PKM2/HIF-1α/DLAT pathway, uncouples glycolysis from mitochondrial metabolism, and enhances sensitivity to copper-induced death.	[231]
Lignin–copper coordinated	Leukemia	As a delivery carrier for tyrosine kinase inhibitors, it releases drugs in response to pH/GSH, and significantly enhances the therapeutic activity of TKIs through the cuproptosis mechanism.	[233]
Ce6@Cu NPs	Glioblastoma	The sonodynamic effect consumes GSH and promotes lipid peroxidation; Cu^2+^ is reduced, downregulating FDX1 and LIAS, and inducing the oligomerization of DLAT.	[234]
siAtox1/ES@OMV	Breast, rectal cancer	Delivering siRNA to knockdown the copper partner protein Atox1 to disrupt copper excretion, combined with Elesclomol (ES) to promote copper influx and mitochondrial accumulation, synergistically induces strong cuproptosis.	[236]
TSF@ES-Cu NPs	Pancreatic cancer	Utilizing the natural RGD peptide and secondary structure for targeting to tumor tissues, ES-Cu is delivered to induce cuproptosis.	[241]
T-T@Cu	Breast cancer	Consumption of GSH and specific release of copper, inhibition of the copper efflux protein ATP7A/B, and exacerbation of mitochondrial copper overload.	[242]
Cu@Fh	Melanoma	Upregulation of MHC-I expression enhances immunogenicity; at the same time, light exposure triggers the release of Fe/Cu ions, and concurrent induction of ferroptosis and cuproptosis occurs.	[243]

## Data Availability

No new data were created or analyzed in this study. Data sharing is not applicable to this article.
